# Supplementary figures and images for: The impact of thermal and auditory unpleasant stimulus on explicit motor imagery in healthy individuals: An experimental study
Source: PLoS One. 2025 Sep 22;20(9):e0321343. doi: 10.1371/journal.pone.0321343 (PMC12453216; doi:10.1371/journal.pone.0321343)

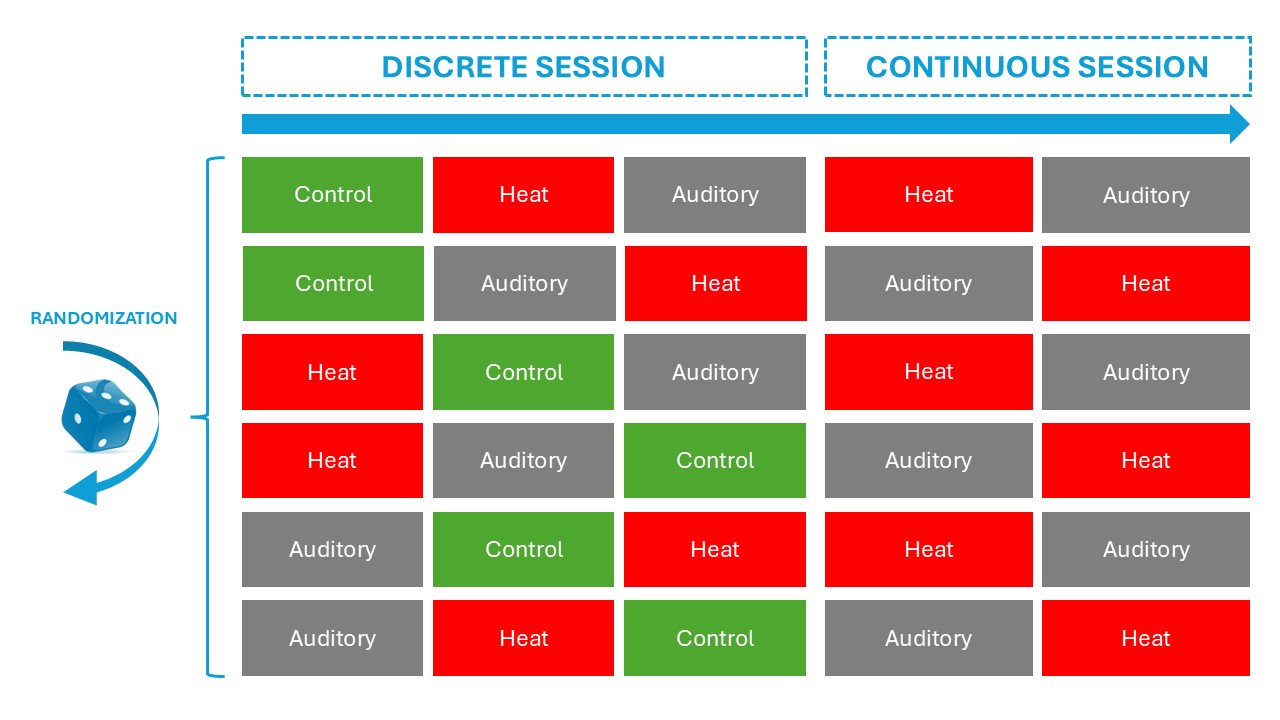

Supplement: S1 Fig — (DOCX) [file pone.0321343.s001.docx]
