# Supplementary material for: The impact of thermal and auditory unpleasant stimulus on explicit motor imagery in healthy individuals: An experimental study
Source: PLoS One. 2025 Sep 22;20(9):e0321343. doi: 10.1371/journal.pone.0321343 (PMC12453216; doi:10.1371/journal.pone.0321343)
Supplement: S1 Table — (DOCX) [file pone.0321343.s002.docx]

|  |  |  |  | **KVIQ scores** | | |  |
| --- | --- | --- | --- | --- | --- | --- | --- |
|  |  |  | **Conditions**  **(n=18)** | **Control** | **Auditory** | **Heat** | **P value** |
| **Discrete Motor Imagery** | | **KVIQ total score** | Median  *(± IQR)* | 7*  *(± 2.75)* | 5*  *(± 3.75)* | 6  *(± 3)* | 0.002 |
|  |  |  | CI (low) | 4.323 | 3 | 4 |  |
|  |  |  | CI (high) | 9.677 | 8.677 | 9.354 |  |
|  |  | **KVIQ-K**  **subscore** | Median  *(± IQR)* | 3.5*  *(± 1)* | 3*^§^  *(± 1)* | 3^§^  *(± 1)* | 0.005 |
|  |  |  | CI (low) | 3 | 2 | 3 |  |
|  |  |  | CI (high) | 4 | 3 | 4 |  |
|  |  | **KVIQ-V**  **subscore** | Median  *(± IQR)* | 3.5*  *(± 1)* | 2*  *(± 2)* | 3  *(± 2)* |  |
|  |  |  | CI (low) | 3 | 2 | 3 | 0.007 |
|  |  |  | CI (high) | 4 | 4 | 4 |  |
| **Continuous Motor Imagery** | | **KVIQ total Score** | Median  *(± IQR)* | 6.5  *(± 3.5)* | 4.5  *(± 3.5)* | 6.0  *(± 2.75)* | 0.645 |
|  |  |  | CI (low) | 4.25 | 4.00 | 4.25 |  |
|  |  |  | CI (high) | 7.75 | 7.50 | 7.00 |  |
|  |  | **KVIQ-K**  **subscore** | Median  *(± IQR)* | 3  *(± 2)* | 2  *(± 1.75)* | 3  *(± 1.75)* | 0.663 |
|  |  |  | CI (low) | 2 | 2 | 2 |  |
|  |  |  | CI (high) | 4 | 3.75 | 3.75 |  |
|  |  | **KVIQ-V**  **subscore** | Median  *(± IQR)* | 3.5  *(± 1.75)* | 2  *(± 1.75)* | 3  *(± 2)* |  |
|  |  |  | CI (low) | 2.25 | 2 | 2 | 0.103 |
|  |  |  | CI (high) | 4 | 3.75 | 4 |  |

KVIQ-K, KVIQ-Kinesthetic subscore. KVIQ-V, KVIQ-Visual subscore. CI, Confidence Interval. IQR, Inter-Quartile Range. *p<0.05 between Control & Auditory conditions, § p<0.05 between Heat & Auditory conditions.
