# Supplementary material for: The impact of thermal and auditory unpleasant stimulus on explicit motor imagery in healthy individuals: An experimental study
Source: PLoS One. 2025 Sep 22;20(9):e0321343. doi: 10.1371/journal.pone.0321343 (PMC12453216; doi:10.1371/journal.pone.0321343)

IMPaiR statistical analysis of Motor Imagery

Exploring the Influence of unpleasant Auditory and Thermal Stimuli on Motor Imagery Abilities: Implications for Pain Models

COHEN-AKNINE Gabriel

2024-08-22

Set path

```
# This function create 2 plots : a boxplot with jitter and a line plot with mean and confidence interva
outlier <- function(x) {
  Q1 <- quantile(x, 0.25)
  Q3 <- quantile(x, 0.75)
  IQR <- Q3 - Q1
  lower_bound <- Q1 - 1.5 * IQR
  upper_bound <- Q3 + 1.5 * IQR

  outliers1 <- x[x < lower_bound | x > upper_bound]

  z_scores <- scale(x)
  outliers2 <- x[abs(z_scores) > 3]
  return(list(outliers1, outliers2))
}
```

Create a functions for outliers counting

Load the dataframe

```
data <- read.csv2("demographics.csv")
```

```
#Replace "," by "." in some columns
data$ContinuWarm_IM_EVA <- as.numeric(gsub(",", ".", data$ContinuWarm_IM_EVA))
data$ContinuSound_Exe_EVA <- as.numeric(gsub(",", ".", data$ContinuSound_Exe_EVA))
data$ContinuSound_IM_EVA <- as.numeric(gsub(",", ".", data$ContinuSound_IM_EVA))

#Rename ID column
colnames(data)[1] <- "ID"
colnames(data)[colnames(data) == "Normal_Exe_EVA"] <- "Control_Exe_VAS"
colnames(data)[colnames(data) == "Normal_IM_EVA"] <- "Control_IM_VAS"
colnames(data)[colnames(data) == "Warm_Exe_EVA"] <- "Warm_Exe_VAS"
```

```
colnames(data)[colnames(data) == "Warm_IM_EVA"] <- "Warm_IM_VAS"
colnames(data)[colnames(data) == "Sound_Exe_EVA"] <- "Sound_Exe_VAS"
colnames(data)[colnames(data) == "Sound_IM_EVA"] <- "Sound_IM_VAS"
colnames(data)[colnames(data) == "ContinuNormal_IM_EVA"] <- "ContinuControl_IM_VAS"
colnames(data)[colnames(data) == "ContinuNormal_Exe_EVA"] <- "ContinuControl_Exe_VAS"
colnames(data)[colnames(data) == "ContinuWarm_IM_EVA"] <- "ContinuWarm_IM_VAS"
colnames(data)[colnames(data) == "ContinuWarm_Exe_EVA"] <- "ContinuWarm_Exe_VAS"
colnames(data)[colnames(data) == "ContinuSound_IM_EVA"] <- "ContinuSound_IM_VAS"
colnames(data)[colnames(data) == "ContinuSound_Exe_EVA"] <- "ContinuSound_Exe_VAS"
colnames(data)[colnames(data) == "Normal_IM_VMI"] <- "Control_IM_VMI"
colnames(data)[colnames(data) == "Normal_IM_KMI"] <- "Control_IM_KMI"
colnames(data)[colnames(data) == "Normal_IM_Total"] <- "Control_IM_Total"
colnames(data)[colnames(data) == "ContinuNormal_IM_VMI"] <- "ContinuControl_IM_VMI"
colnames(data)[colnames(data) == "ContinuNormal_IM_KMI"] <- "ContinuControl_IM_KMI"
colnames(data)[colnames(data) == "ContinuNormal_IM_Total"] <- "ContinuControl_IM_Total"
```

Demographic analysis

The small sample size of this study will be analyzed using non-parametric tests. Statistics will be presented as median and interquartile range (IQR).

Demographic description

```
# calculate the median and IQR and CI for each variable

# Calcul des statistiques descriptives
# Calcul des statistiques descriptives
summary_stats <- data.frame(
  Variable = c("Age", "Size", "Mass", "BMI", "IPAQ_MET", "IPAQ_inactivity", "KVIQ_V", "KVIQ_K", "Volume
  Median = c(
    ifelse("Age" %in% names(data), median(data$Age, na.rm = TRUE), NA),
    ifelse("Size" %in% names(data), median(data$Size, na.rm = TRUE), NA),
    ifelse("Mass" %in% names(data), median(data$Mass, na.rm = TRUE), NA),
    ifelse("BMI" %in% names(data), median(data$BMI, na.rm = TRUE), NA),
    ifelse("IPAQ_MET" %in% names(data), median(data$IPAQ_MET, na.rm = TRUE), NA),
    ifelse("IPAQ_inactivity" %in% names(data), median(data$IPAQ_inactivity, na.rm = TRUE), NA),
    ifelse("KVIQ_V" %in% names(data), median(data$KVIQ_V, na.rm = TRUE), NA),
    ifelse("KVIQ_K" %in% names(data), median(data$KVIQ_K, na.rm = TRUE), NA),
    ifelse("Volume" %in% names(data), median(data$Volume, na.rm = TRUE), NA),
    ifelse("Temperature" %in% names(data), median(data$Temperature, na.rm = TRUE), NA),
    ifelse("EHI" %in% names(data), median(data$EHI, na.rm = TRUE), NA)
  ),
  IQR = c(
    ifelse("Age" %in% names(data), IQR(data$Age, na.rm = TRUE), NA),
    ifelse("Size" %in% names(data), IQR(data$Size, na.rm = TRUE), NA),
    ifelse("Mass" %in% names(data), IQR(data$Mass, na.rm = TRUE), NA),
    ifelse("BMI" %in% names(data), IQR(data$BMI, na.rm = TRUE), NA),
    ifelse("IPAQ_MET" %in% names(data), IQR(data$IPAQ_MET, na.rm = TRUE), NA),
    ifelse("IPAQ_inactivity" %in% names(data), IQR(data$IPAQ_inactivity, na.rm = TRUE), NA),
    ifelse("KVIQ_V" %in% names(data), IQR(data$KVIQ_V, na.rm = TRUE), NA),
```

```
    ifelse("KVIQ_K" %in% names(data), IQR(data$KVIQ_K, na.rm = TRUE), NA),
    ifelse("Volume" %in% names(data), IQR(data$Volume, na.rm = TRUE), NA),
    ifelse("Temperature" %in% names(data), IQR(data$Temperature, na.rm = TRUE), NA),
    ifelse("EHI" %in% names(data), IQR(data$EHI, na.rm = TRUE), NA)
  ),
  CI_upper = c(
    ifelse("Age" %in% names(data), quantile(data$Age, probs = 0.75, na.rm = TRUE) + 1.96 * IQR(data$Age
    ifelse("Size" %in% names(data), quantile(data$Size, probs = 0.75, na.rm = TRUE) + 1.96 * IQR(data$Si
    ifelse("Mass" %in% names(data), quantile(data$Mass, probs = 0.75, na.rm = TRUE) + 1.96 * IQR(data$Ma
    ifelse("BMI" %in% names(data), quantile(data$BMI, probs = 0.75, na.rm = TRUE) + 1.96 * IQR(data$BMI
    ifelse("IPAQ_MET" %in% names(data), quantile(data$IPAQ_MET, probs = 0.75, na.rm = TRUE) + 1.96 * IQ
    ifelse("IPAQ_inactivity" %in% names(data), quantile(data$IPAQ_inactivity, probs = 0.75, na.rm = TRU
    ifelse("KVIQ_V" %in% names(data), quantile(data$KVIQ_V, probs = 0.75, na.rm = TRUE) + 1.96 * IQR(da
    ifelse("KVIQ_K" %in% names(data), quantile(data$KVIQ_K, probs = 0.75, na.rm = TRUE) + 1.96 * IQR(da
    ifelse("Volume" %in% names(data), quantile(data$Volume, probs = 0.75, na.rm = TRUE) + 1.96 * IQR(da
    ifelse("Temperature" %in% names(data), quantile(data$Temperature, probs = 0.75, na.rm = TRUE) + 1.9
    ifelse("EHI" %in% names(data), quantile(data$EHI, probs = 0.75, na.rm = TRUE) + 1.96 * IQR(data$EHI
  ),
  CI_lower = c(
    ifelse("Age" %in% names(data), quantile(data$Age, probs = 0.25, na.rm = TRUE) - 1.96 * IQR(data$Age
    ifelse("Size" %in% names(data), quantile(data$Size, probs = 0.25, na.rm = TRUE) - 1.96 * IQR(data$Si
    ifelse("Mass" %in% names(data), quantile(data$Mass, probs = 0.25, na.rm = TRUE) - 1.96 * IQR(data$Ma
    ifelse("BMI" %in% names(data), quantile(data$BMI, probs = 0.25, na.rm = TRUE) - 1.96 * IQR(data$BMI
    ifelse("IPAQ_MET" %in% names(data), quantile(data$IPAQ_MET, probs = 0.25, na.rm = TRUE) - 1.96 * IQ
    ifelse("IPAQ_inactivity" %in% names(data), quantile(data$IPAQ_inactivity, probs = 0.25, na.rm = TRU
    ifelse("KVIQ_V" %in% names(data), quantile(data$KVIQ_V, probs = 0.25, na.rm = TRUE) - 1.96 * IQR(da
    ifelse("KVIQ_K" %in% names(data), quantile(data$KVIQ_K, probs = 0.25, na.rm = TRUE) - 1.96 * IQR(da
    ifelse("Volume" %in% names(data), quantile(data$Volume, probs = 0.25, na.rm = TRUE) - 1.96 * IQR(da
    ifelse("Temperature" %in% names(data), quantile(data$Temperature, probs = 0.25, na.rm = TRUE) - 1.9
    ifelse("EHI" %in% names(data), quantile(data$EHI, probs = 0.25, na.rm = TRUE) - 1.96 * IQR(data$EHI
  ),
  stringsAsFactors = FALSE
)
```

```
# Print the summary statistics
kable(summary_stats, caption = "Descriptive Statistics")
```

Table 1: Descriptive Statistics

Variable	Median	IQR	CI_upper	CI_lower
Age	23.00	3.500	27.11692	20.38308
Size	177.50	11.500	185.31273	163.18727
Mass	69.50	19.000	87.77755	51.22245
BMI	22.55	4.150	26.49220	18.50780
IPAQ_MET	5003.50	3633.750	9426.95685	2435.79315
IPAQ_inactivity	2310.00	1260.000	3522.09030	1097.90970
KVIQ_V	19.00	2.000	20.92395	17.07605
KVIQ_K	18.00	2.000	19.92395	16.07605
Volume	81.00	8.000	87.44581	72.05419
Temperature	46.00	1.875	47.74121	44.13379
EHI	80.00	25.000	111.88425	63.11575

```r
# create a word document
doc <- read_docx() %>%
  body_add_table(summary_stats, style = "table_template")

# export the word in .docx format
print(doc, target = "summary_stats.docx")
```

Primary Outcome :

Motor Imagery during Discrete Motion session

Total Score

Create a dataframe to analyze total score for motor imagery between conditions

```r
# Create a dataframe for the Motor Imagery Total Score
MI_Total <- data %>%
  select(ID, Control_IM_Total, Warm_IM_Total, Sound_IM_Total) %>%
  pivot_longer(
    cols = c(Control_IM_Total, Warm_IM_Total, Sound_IM_Total),
    names_to = "condition",
    values_to = "MI_TotalScore"
  ) %>%
  mutate(condition = case_when(
    condition == "Control_IM_Total" ~ "Control",
    condition == "Warm_IM_Total" ~ "Heat",
    condition == "Sound_IM_Total" ~ "Auditory"
  ))

# Detect outliers
outlier(MI_Total$MI_TotalScore)
```

```
## [[1]]
## numeric(0)
##
## [[2]]
## numeric(0)
```

Visualize the data

```r
MI_Total$condition <- factor(MI_Total$condition, levels = c("Control", "Heat", "Auditory"))

compare_means(MI_TotalScore ~ condition, data = MI_Total)
```

```
## # A tibble: 3 x 8
##   .y.           group1  group2        p p.adj p.format p.signif method
##   <chr>         <chr>   <chr>     <dbl> <dbl> <chr>    <chr>    <chr>
## 1 MI_TotalScore Control Heat     0.399   0.4  0.399    ns       Wilcoxon
## 2 MI_TotalScore Control Auditory 0.0429  0.13 0.043    *        Wilcoxon
## 3 MI_TotalScore Heat    Auditory 0.133   0.27 0.133    ns       Wilcoxon
```

```r
my_comparisons <- list( c("Control", "Heat"), c("Control", "Auditory"), c("Heat", "Auditory") )

PlotA <- ggplot(MI_Total, aes(x = condition, y = MI_TotalScore, fill = condition)) +
  geom_boxplot(alpha = 0.7) +
  geom_jitter(aes(fill = condition), position = position_jitter(0.1), size = 2, alpha = 0.5) +
  labs(title = "Motor Imagery Total Score during discrete session", x = "Condition", y = "MI Total Scor
  theme_minimal() +
  theme(plot.title = element_text(hjust = 0.5))+
  scale_fill_manual(values = c("Control" = "green", "Heat" = "red", "Auditory" = "grey"))+
  ylim(0, 5)

PlotA
```

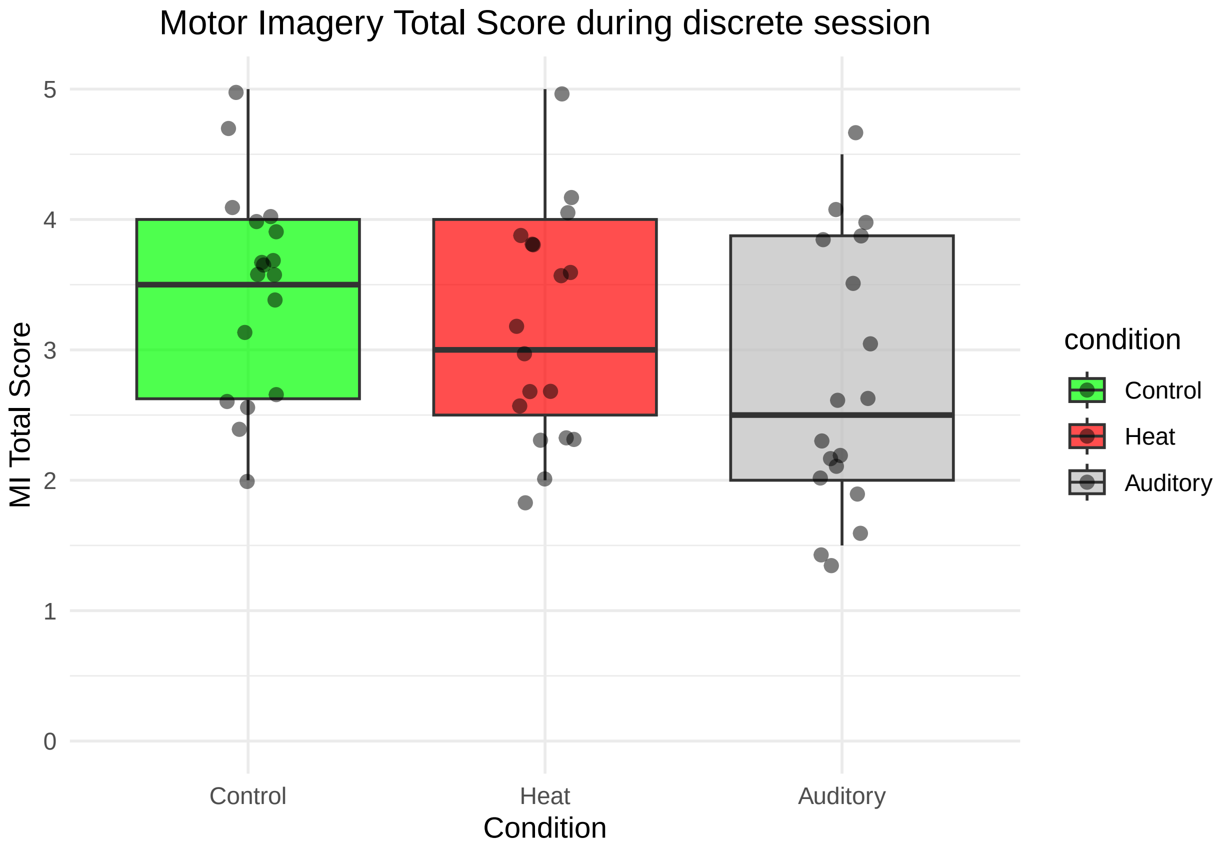

Based on the QQ plot and Shapiro test interpretation, we can conclude that the residuals are normally distributed for the Total score. Parametric test will be used for the analysis.

Statistical analysis

```r
friedman_test(MI_Total, MI_TotalScore ~ condition | ID)
```

```
## # A tibble: 1 x 6
##   .y.               n statistic    df       p method
## * <chr>         <int>     <dbl> <dbl>   <dbl> <chr>
## 1 MI_TotalScore    18      12.0     2 0.00243 Friedman test
```

The Friedman test show a statistically significant effect of condition on MI Total Score ($p < 0.001$).

```
#Post hoc analysis
wilcox_test(MI_Total, MI_TotalScore ~ condition, paired = TRUE, p.adjust.method = "bonferroni")
```

```
## # A tibble: 3 x 9
##   .y.           group1  group2      n1    n2 statistic     p p.adj p.adj.signif
## * <chr>         <chr>   <chr>    <int> <int>     <dbl> <dbl> <dbl> <chr>
## 1 MI_TotalScore Control Heat        18    18      43   0.112 0.336 ns
## 2 MI_TotalScore Control Auditory    18    18     111   0.003 0.01  *
## 3 MI_TotalScore Heat    Auditory    18    18      77.5 0.025 0.074 ns
```

```
#Effect size
wilcox_effsize(MI_Total, MI_TotalScore ~ condition, paired = TRUE)
```

```
## # A tibble: 3 x 7
##   .y.           group1  group2   effsize    n1    n2 magnitude
## * <chr>         <chr>   <chr>      <dbl> <int> <int> <ord>
## 1 MI_TotalScore Control Heat       0.343    18    18 moderate
## 2 MI_TotalScore Control Auditory   0.703    18    18 large
## 3 MI_TotalScore Heat    Auditory   0.571    18    18 large
```

Post hoc analysis shows a significant difference between the Control and Auditory conditions ($p = 0.010$) with a large effect size (-0.702).

Summary of MI Total score

```
MI_Total %>%
  group_by(condition) %>%
  summarise(
    median = median(MI_TotalScore, na.rm = TRUE),
    median_CI_low = quantile(MI_TotalScore, probs = 0.5 - 1.96 * sqrt(0.25 / n()), na.rm = TRUE),
    median_CI_high = quantile(MI_TotalScore, probs = 0.5 + 1.96 * sqrt(0.25 / n()), na.rm = TRUE),
    IQR = IQR(MI_TotalScore, na.rm = TRUE),
    IQR_CI_low = quantile(MI_TotalScore, probs = 0.25 - 1.96 * sqrt(0.25 / n()), na.rm = TRUE),
    IQR_CI_high = quantile(MI_TotalScore, probs = 0.75 + 1.96 * sqrt(0.25 / n()), na.rm = TRUE),

    n = n()
  )
```

```
## # A tibble: 3 x 8
##   condition median median_CI_low median_CI_high   IQR IQR_CI_low IQR_CI_high
##   <fct>      <dbl>         <dbl>          <dbl> <dbl>      <dbl>       <dbl>
## 1 Control      3.5          2.79           4     1.38       2.16        4.84
## 2 Heat         3            2.5            4     1.5        2           4.68
## 3 Auditory     2.5          2              3.71  1.88       1.5         4.34
## # i 1 more variable: n <int>
```

Conclusion

The results of the analysis indicate that the auditory unpleasant stimulus moderately to highly decreases MI abilities compared to heat and Control conditions.

#Secondary outcome : Motor Imagery during Continuous Motion session

Total Score

```
# Create a dataframe for the Motor Imagery Total Score
ContinuMI_Total <- data %>%
  select(ID, ContinuControl_IM_Total, ContinuWarm_IM_Total, ContinuSound_IM_Total) %>%
  pivot_longer(
    cols = c(ContinuControl_IM_Total, ContinuWarm_IM_Total, ContinuSound_IM_Total),
    names_to = "condition",
    values_to = "ContinuMI_TotalScore"
  ) %>%
  mutate(condition = case_when(
    condition == "ContinuControl_IM_Total" ~ "Control",
    condition == "ContinuWarm_IM_Total" ~ "Heat",
    condition == "ContinuSound_IM_Total" ~ "Auditory"
  ))

# Detect outliers
outlier(ContinuMI_Total$ContinuMI_TotalScore)
```

```
## [[1]]
## numeric(0)
##
## [[2]]
## numeric(0)
```

Visualize the data

```
ContinuMI_Total$condition <- factor(MI_Total$condition, levels = c("Control", "Heat", "Auditory"))

ggplot(ContinuMI_Total, aes(x = condition, y = ContinuMI_TotalScore, fill = condition)) +
  geom_boxplot(alpha = 0.7) +
  geom_jitter(aes(fill = condition), position = position_jitter(0.1), size = 2, alpha = 0.5)+
  labs(title = "Motor Imagery Total Score during continuous session", x = "Condition", y = "MI Total Sco
  theme_minimal() +
  theme(plot.title = element_text(hjust = 0.5))+
  scale_fill_manual(values = c("Control" = "green", "Heat" = "red", "Auditory" = "grey"))
```

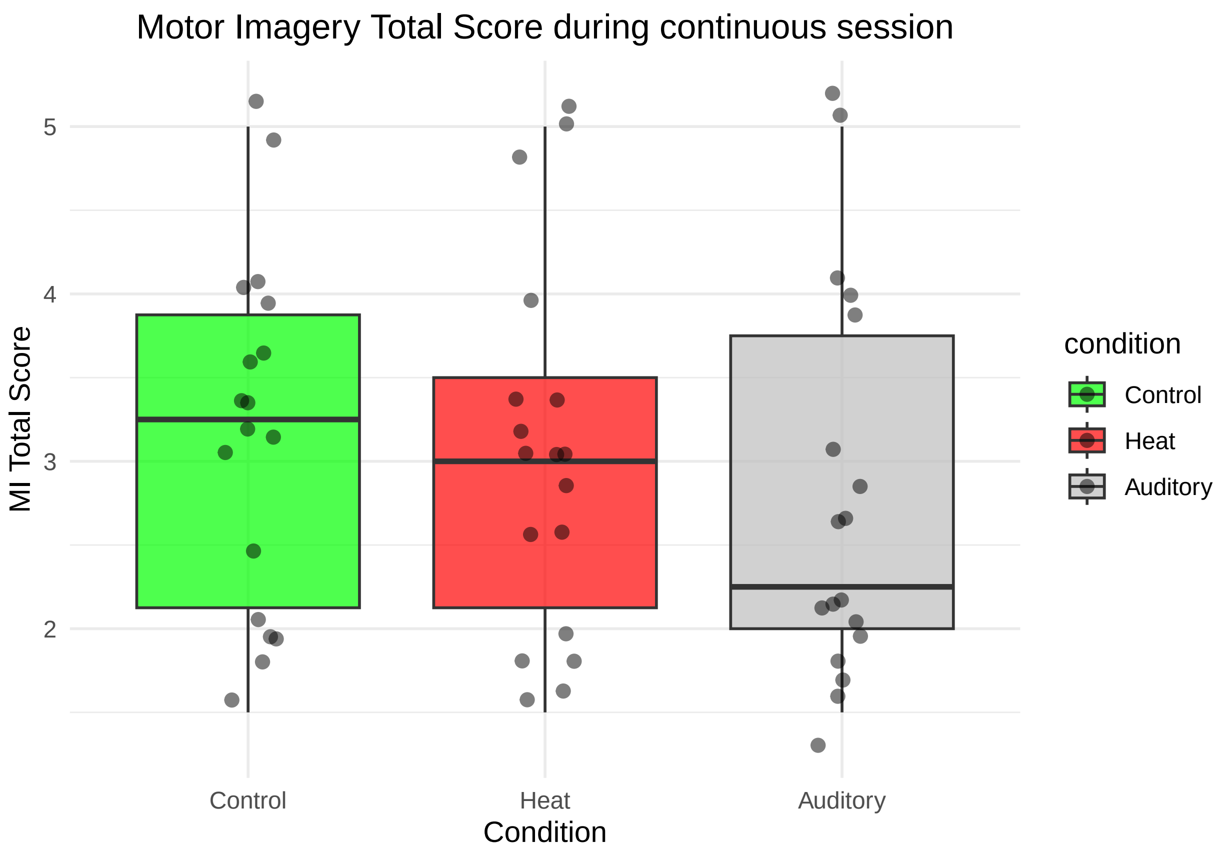

Statistical analysis

```
friedman_test(ContinuMI_Total, ContinuMI_TotalScore ~ condition | ID)
```

```
## # A tibble: 1 x 6
##   .y.                      n statistic    df     p method
## * <chr>                <int>     <dbl> <dbl> <dbl> <chr>
## 1 ContinuMI_TotalScore    18     0.875     2 0.646 Friedman test
```

The Friedman test shown that there is no statistical differences between groups.

```
ContinuMI_Total %>%
  group_by(condition) %>%
  summarise(
    median = median(ContinuMI_TotalScore, na.rm = TRUE),
    IQR = IQR(ContinuMI_TotalScore, na.rm = TRUE),
    CI_low = quantile(ContinuMI_TotalScore, probs = 0.25, na.rm = TRUE),
    CI_high = quantile(ContinuMI_TotalScore, probs = 0.75, na.rm = TRUE),
    n = n()
  )
```

Summary of MI Total score

```
## # A tibble: 3 x 6
##   condition median   IQR CI_low CI_high     n
##   <fct>      <dbl> <dbl>  <dbl>   <dbl> <int>
## 1 Control     3.25  1.75   2.12    3.88    18
## 2 Heat        3     1.38   2.12    3.5     18
## 3 Auditory    2.25  1.75   2       3.75    18
```

Complementary analysis

KMI and VMI subscores during Discrete Motion session

```r
# KMI and VMI score

# Create a dataframe for the Motor Imagery KMI Score
MI_KMI <- data %>%
  select(ID, Control_IM_KMI, Warm_IM_KMI, Sound_IM_KMI) %>%
  pivot_longer(
    cols = c(Control_IM_KMI, Warm_IM_KMI, Sound_IM_KMI),
    names_to = "condition",
    values_to = "MI_KMI"
  ) %>%
  mutate(condition = case_when(
    condition == "Control_IM_KMI" ~ "Control",
    condition == "Warm_IM_KMI" ~ "Heat",
    condition == "Sound_IM_KMI" ~ "Auditory"
  ))

# Detect outliers
outlier(MI_KMI$MI_KMI)
```

```
## [[1]]
## integer(0)
##
## [[2]]
## integer(0)
```

```r
# Create a dataframe for the Motor Imagery VMI Score
MI_VMI <- data %>%
  select(ID, Control_IM_VMI, Warm_IM_VMI, Sound_IM_VMI) %>%
  pivot_longer(
    cols = c(Control_IM_VMI, Warm_IM_VMI, Sound_IM_VMI),
    names_to = "condition",
    values_to = "MI_VMI"
  ) %>%
  mutate(condition = case_when(
    condition == "Control_IM_VMI" ~ "Control",
    condition == "Warm_IM_VMI" ~ "Heat",
    condition == "Sound_IM_VMI" ~ "Auditory"
```

```r
  ))

# Detect outliers
outlier(MI_VMI$MI_VMI)
```

```
## [[1]]
## integer(0)
##
## [[2]]
## integer(0)
```

Visualize the data

```r
MI_KMI$condition <- factor(MI_KMI$condition, levels = c("Control", "Heat", "Auditory"))

compare_means(MI_KMI ~ condition, data = MI_KMI)
```

For KMI subscore

```
## # A tibble: 3 x 8
##   .y.    group1  group2          p p.adj p.format p.signif method
##   <chr>  <chr>   <chr>       <dbl> <dbl> <chr>    <chr>    <chr>
## 1 MI_KMI Control Heat     0.589    0.59  0.589    ns       Wilcoxon
## 2 MI_KMI Control Auditory 0.0289   0.087 0.029    *        Wilcoxon
## 3 MI_KMI Heat    Auditory 0.103    0.21  0.103    ns       Wilcoxon
```

```r
my_comparisons <- list( c("Control", "Heat"), c("Control", "Auditory"), c("Heat", "Auditory") )

PlotB <- ggplot(MI_KMI, aes(x = condition, y = MI_KMI, fill = condition)) +
  geom_boxplot(alpha = 0.7) +
  geom_jitter(aes(fill = condition), position = position_jitter(0.1), size = 2, alpha = 0.5)+
  labs(title = "Motor Imagery KMI Score during discrete motion session", x = "Condition", y = "KMI Scor
  theme_minimal() +
  theme(plot.title = element_text(hjust = 0.5))+
  scale_fill_manual(values = c("Control" = "green", "Heat" = "red", "Auditory" = "grey"))+
  ylim(0, 5)

PlotB
```

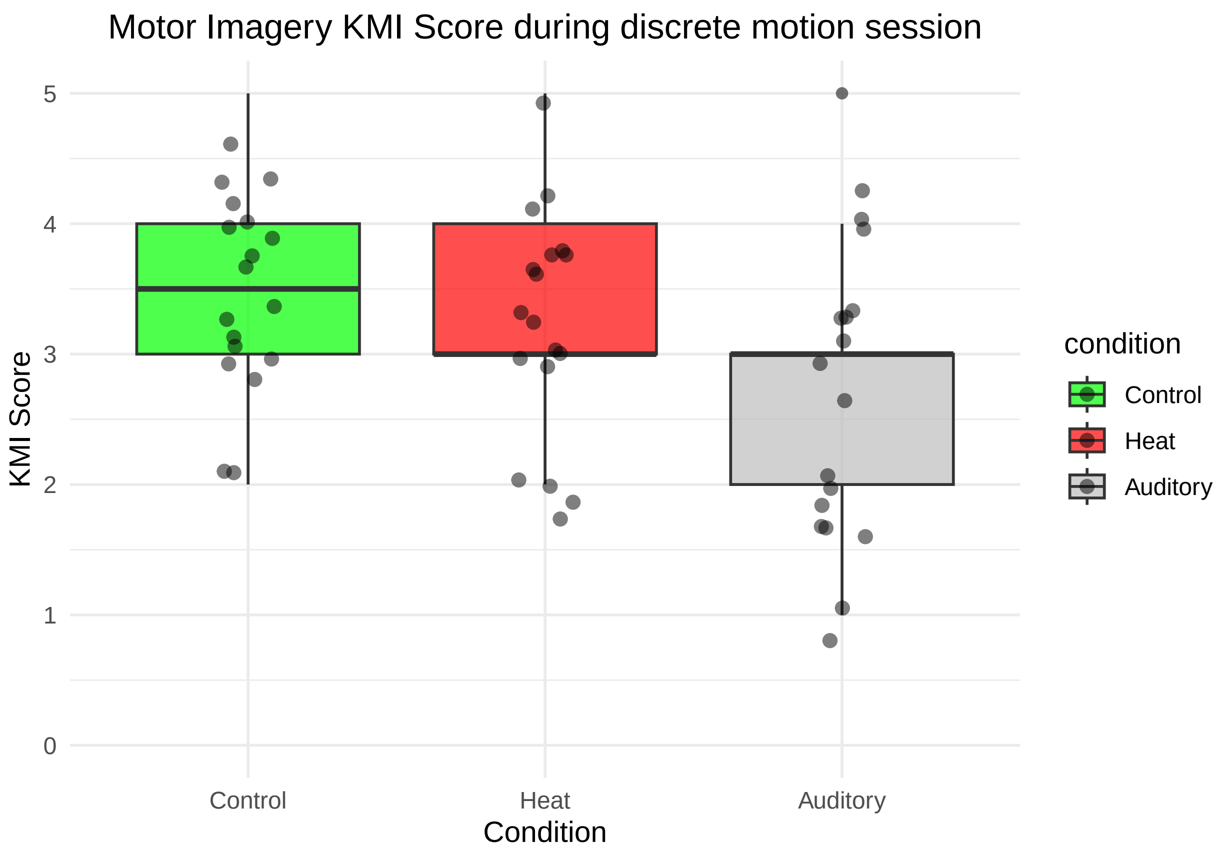


```
MI_VMI$condition <- factor(MI_VMI$condition, levels = c("Control", "Heat", "Auditory"))

compare_means(MI_VMI ~ condition, data = MI_VMI)
```

For VMI subscore

```
## # A tibble: 3 x 8
##   .y.    group1  group2        p p.adj p.format p.signif method
##   <chr>  <chr>   <chr>     <dbl> <dbl> <chr>    <chr>    <chr>
## 1 MI_VMI Control Heat     0.307   0.61 0.307    ns       Wilcoxon
## 2 MI_VMI Control Auditory 0.0641  0.19 0.064    ns       Wilcoxon
## 3 MI_VMI Heat    Auditory 0.310   0.61 0.310    ns       Wilcoxon
```

```
my_comparisons <- list( c("Control", "Heat"), c("Control", "Auditory"), c("Heat", "Auditory") )

PlotC <- ggplot(MI_VMI, aes(x = condition, y = MI_VMI, fill = condition)) +
  geom_boxplot(alpha = 0.7) +
  geom_jitter(aes(fill = condition), position = position_jitter(0.1), size = 2, alpha = 0.5) +
  labs(title = "Motor Imagery VMI Score", x = "Condition", y = "VMI Score") +
  theme_minimal() +
  theme(plot.title = element_text(hjust = 0.5))+
  scale_fill_manual(values = c("Control" = "green", "Heat" = "red", "Auditory" = "grey"))+
```

```r
  ylim(0, 5)

PlotC
```

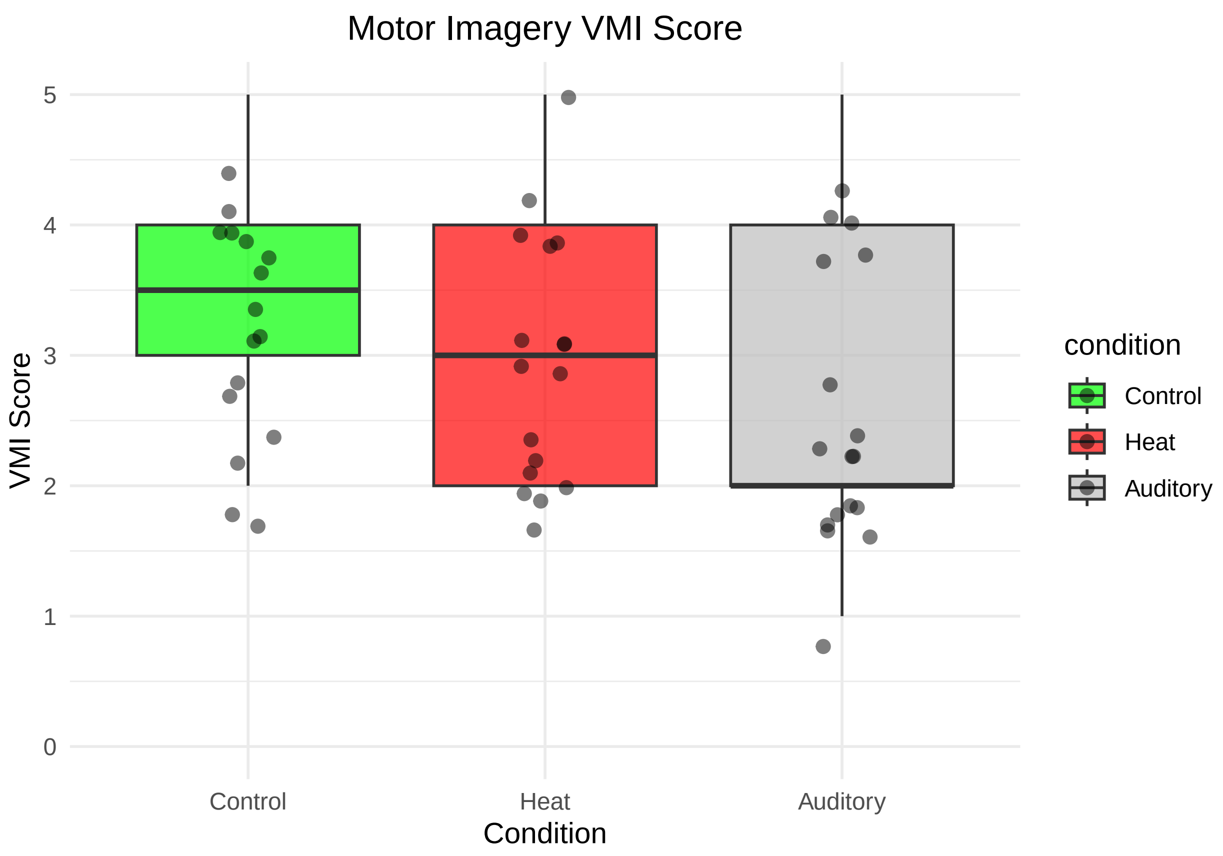

Statistical analysis

```r
friedman_test(MI_KMI, MI_KMI ~ condition | ID)
```

For the KMI score subscore

```
## # A tibble: 1 x 6
##   .y.        n statistic    df       p method
## * <chr>  <int>     <dbl> <dbl>   <dbl> <chr>
## 1 MI_KMI    18      10.7     2 0.00479 Friedman test
```

The friedman test show a statistically significant effect of condition on KMI Score ($p = 0.0048$).

```r
friedman_test(MI_VMI, MI_VMI ~ condition | ID)
```

For the VMI score

```
## # A tibble: 1 x 6
##   .y.        n statistic    df       p method
## * <chr>  <int>     <dbl> <dbl>   <dbl> <chr>
## 1 MI_VMI    18      10.1     2 0.00657 Friedman test
```

The friedman test show a statistically significant effect of condition on KMI Score (p = 0.0066).

```r
# Post hoc analysis
wilcox_test(MI_KMI, MI_KMI ~ condition, paired = TRUE, p.adjust.method = "bonferroni")
```

Post hoc analysis for KMI subscore

```
## # A tibble: 3 x 9
##   .y.    group1  group2      n1    n2 statistic     p p.adj p.adj.signif
## * <chr>  <chr>   <chr>    <int> <int>     <dbl> <dbl> <dbl> <chr>
## 1 MI_KMI Control Heat        18    18      11   0.408 1     ns
## 2 MI_KMI Control Auditory    18    18      80   0.012 0.037 *
## 3 MI_KMI Heat    Auditory    18    18      50.5 0.015 0.045 *
```

```r
#Effect size
wilcox_effsize(MI_KMI, MI_KMI ~ condition, paired = TRUE)
```

```
## # A tibble: 3 x 7
##   .y.    group1  group2   effsize    n1    n2 magnitude
## * <chr>  <chr>   <chr>      <dbl> <int> <int> <ord>
## 1 MI_KMI Control Heat       0.131    18    18 small
## 2 MI_KMI Control Auditory   0.605    18    18 large
## 3 MI_KMI Heat    Auditory   0.601    18    18 large
```

The post hoc analysis shows a significant difference between the Normal and Auditory conditions (p = 0.037) with a large effect size (0.605), and between the Heat and Auditory condition (p = 0.045) with a large effect size (0.6). However, there is no significant difference between the Control and Heat conditions (p = 1).

```r
# Post hoc analysis
wilcox_test(MI_VMI, MI_VMI ~ condition, paired = TRUE, p.adjust.method = "bonferroni")
```

Post hoc analysis for KMI subscore

```
## # A tibble: 3 x 9
##   .y.    group1  group2      n1    n2 statistic     p p.adj p.adj.signif
## * <chr>  <chr>   <chr>    <int> <int>     <dbl> <dbl> <dbl> <chr>
## 1 MI_VMI Control Heat        18    18        36 0.095 0.286 ns
## 2 MI_VMI Control Auditory    18    18        45 0.007 0.021 *
## 3 MI_VMI Heat    Auditory    18    18        36 0.095 0.286 ns
```

```r
#Effect size
wilcox_effsize(MI_VMI, MI_VMI ~ condition, paired = TRUE)
```

```
## # A tibble: 3 x 7
##   .y.    group1  group2   effsize    n1    n2 magnitude
## * <chr>  <chr>   <chr>      <dbl> <int> <int> <ord>
## 1 MI_VMI Control Heat       0.402    18    18 moderate
## 2 MI_VMI Control Auditory   0.699    18    18 large
## 3 MI_VMI Heat    Auditory   0.402    18    18 moderate
```

The post hoc analysis shows a significant difference between the Control and Auditory conditions ($p = 0.021$) with a large effect size (0.699).However, there is no significant difference between the Control and Heat conditions ($p = 0.286$), and between the Heat and Auditory condition ($p = 0.286$).

Summary

```r
MI_KMI %>%
  group_by(condition) %>%
  summarise(
    median = median(MI_KMI, na.rm = TRUE),
    IQR = IQR(MI_KMI, na.rm = TRUE),
    CI_low = quantile(MI_KMI, probs = 0.25, na.rm = TRUE),
    CI_high = quantile(MI_KMI, probs = 0.75, na.rm = TRUE),
    n = n()
  )
```

Summary of KMI subsubscore

```
## # A tibble: 3 x 6
##   condition median   IQR CI_low CI_high     n
##   <fct>      <dbl> <dbl>  <dbl>   <dbl> <int>
## 1 Control      3.5     1      3       4    18
## 2 Heat         3       1      3       4    18
## 3 Auditory     3       1      2       3    18
```

```r
MI_VMI %>%
  group_by(condition) %>%
  summarise(
    median = median(MI_VMI, na.rm = TRUE),
    IQR = IQR(MI_VMI, na.rm = TRUE),
    CI_low = quantile(MI_VMI, probs = 0.25, na.rm = TRUE),
    CI_high = quantile(MI_VMI, probs = 0.75, na.rm = TRUE),
    n = n()
  )
```

Summary of KMI subscore

```
## # A tibble: 3 x 6
##   condition median   IQR CI_low CI_high     n
##   <fct>      <dbl> <dbl>  <dbl>   <dbl> <int>
## 1 Control      3.5     1      3       4    18
## 2 Heat         3       2      2       4    18
## 3 Auditory     2       2      2       4    18
```

Combine the plots during discrete session

```
ggarrange(
  PlotA +
    labs(title = "KVIQ Total Score") +
    theme(axis.title.y = element_blank()) + ylim(0,5.5),
  PlotB +
    labs(title = "KVIQ-K Score") +
    theme(axis.title.y = element_blank())+ ylim(0,5.5),
  PlotC +
    labs(title = "KVIQ-V Score") +
    theme(axis.title.y = element_blank())+ ylim(0,5.5),
  labels = c("A", "B", "C"),
  ncol = 3,
  nrow = 1,
  common.legend = TRUE,
  legend = "bottom"
)
```

```
## Scale for y is already present.
## Adding another scale for y, which will replace the existing scale.
## Scale for y is already present.
## Adding another scale for y, which will replace the existing scale.
## Scale for y is already present.
## Adding another scale for y, which will replace the existing scale.
```

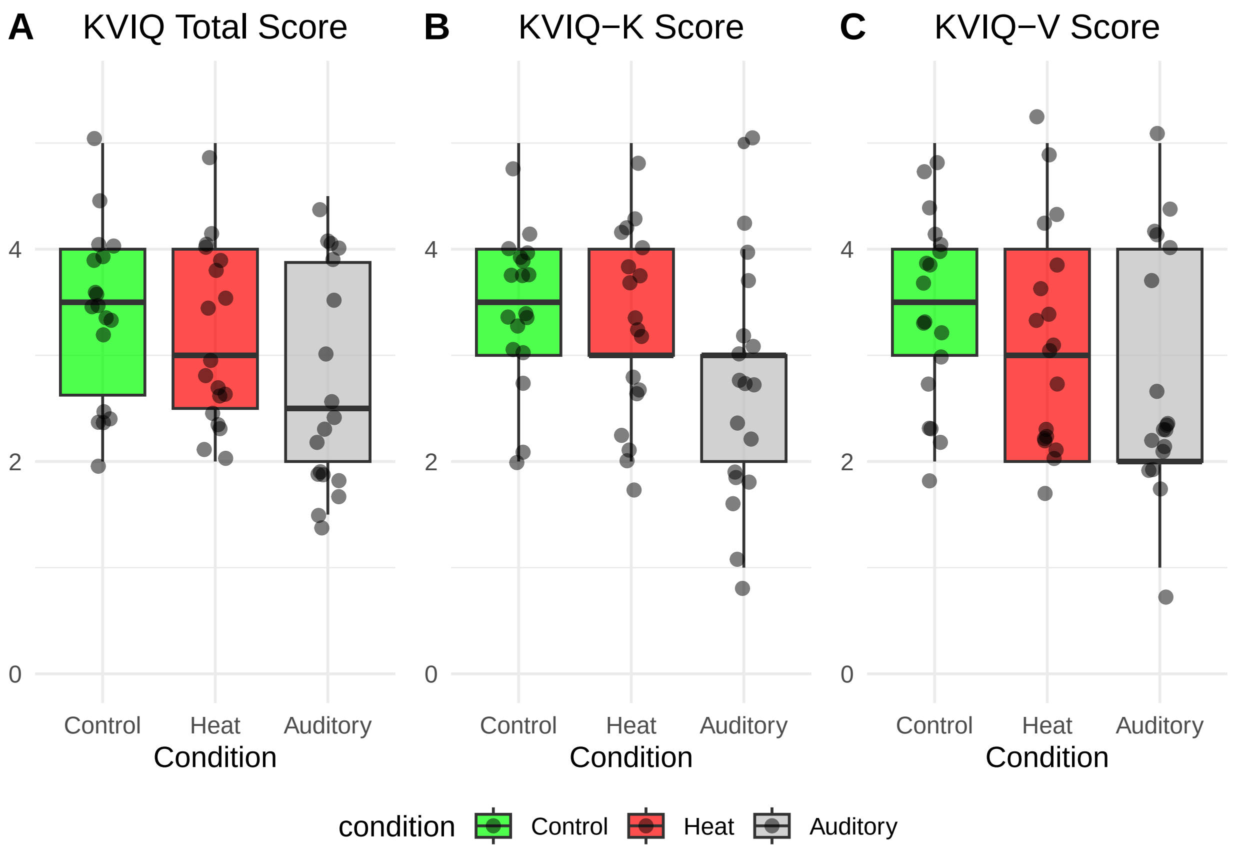

KMI and VMI subscores during continuous Motion session

KMI and VMI Sub-Scores

```
# Create a dataframe for the Motor Imagery KMI Score
ContinuMI_KMI <- data %>%
  select(ID, ContinuControl_IM_KMI, ContinuWarm_IM_KMI, ContinuSound_IM_KMI) %>%
  pivot_longer(
    cols = c(ContinuControl_IM_KMI, ContinuWarm_IM_KMI, ContinuSound_IM_KMI),
    names_to = "condition",
    values_to = "ContinuMI_KMI"
  ) %>%
  mutate(condition = case_when(
    condition == "ContinuControl_IM_KMI" ~ "Control",
    condition == "ContinuWarm_IM_KMI" ~ "Heat",
    condition == "ContinuSound_IM_KMI" ~ "Auditory"
  ))

# Detect outliers
outlier(ContinuMI_KMI$ContinuMI_KMI)
```

```
## [[1]]
## integer(0)
```

```
##
## [[2]]
## integer(0)
```

```
# Create a dataframe for the Motor Imagery Total Score
ContinuMI_VMI <- data %>%
  select(ID, ContinuControl_IM_VMI, ContinuWarm_IM_VMI, ContinuSound_IM_VMI) %>%
  pivot_longer(
    cols = c(ContinuControl_IM_VMI, ContinuWarm_IM_VMI, ContinuSound_IM_VMI),
    names_to = "condition",
    values_to = "ContinuMI_VMI"
  ) %>%
  mutate(condition = case_when(
    condition == "ContinuControl_IM_VMI" ~ "Control",
    condition == "ContinuWarm_IM_VMI" ~ "Heat",
    condition == "ContinuSound_IM_VMI" ~ "Auditory"
  ))

# Detect outliers
outlier(ContinuMI_VMI$ContinuMI_VMI)
```

```
## [[1]]
## integer(0)
##
## [[2]]
## integer(0)
```

Visualize the data

```


ContinuMI_KMI$condition <- factor(ContinuMI_KMI$condition, levels = c("Control", "Heat", "Auditory"))

compare_means(ContinuMI_KMI ~ condition, data = ContinuMI_KMI)
```

For KMI subscore

```
## # A tibble: 3 x 8
##   .y.           group1  group2       p p.adj p.format p.signif method
##   <chr>         <chr>   <chr>    <dbl> <dbl> <chr>    <chr>    <chr>
## 1 ContinuMI_KMI Control Heat     0.657  1    0.66     ns       Wilcoxon
## 2 ContinuMI_KMI Control Auditory 0.295  0.88 0.29     ns       Wilcoxon
## 3 ContinuMI_KMI Heat    Auditory 0.621  1    0.62     ns       Wilcoxon
```

```
my_comparisons <- list( c("Control", "Heat"), c("Control", "Auditory"), c("Heat", "Auditory") )

ggplot(ContinuMI_KMI, aes(x = condition, y = ContinuMI_KMI, fill = condition)) +
  geom_boxplot(alpha = 0.7) +
  geom_jitter(aes(fill = condition), position = position_jitter(0.1), size = 2, alpha = 0.5) +
  labs(title = "Motor Imagery KMI Score during continuous session", x = "Condition", y = "KMI Score") +
  theme_minimal() +
  theme(plot.title = element_text(hjust = 0.5))+
  scale_fill_manual(values = c("Control" = "green", "Heat" = "red", "Auditory" = "grey"))
```

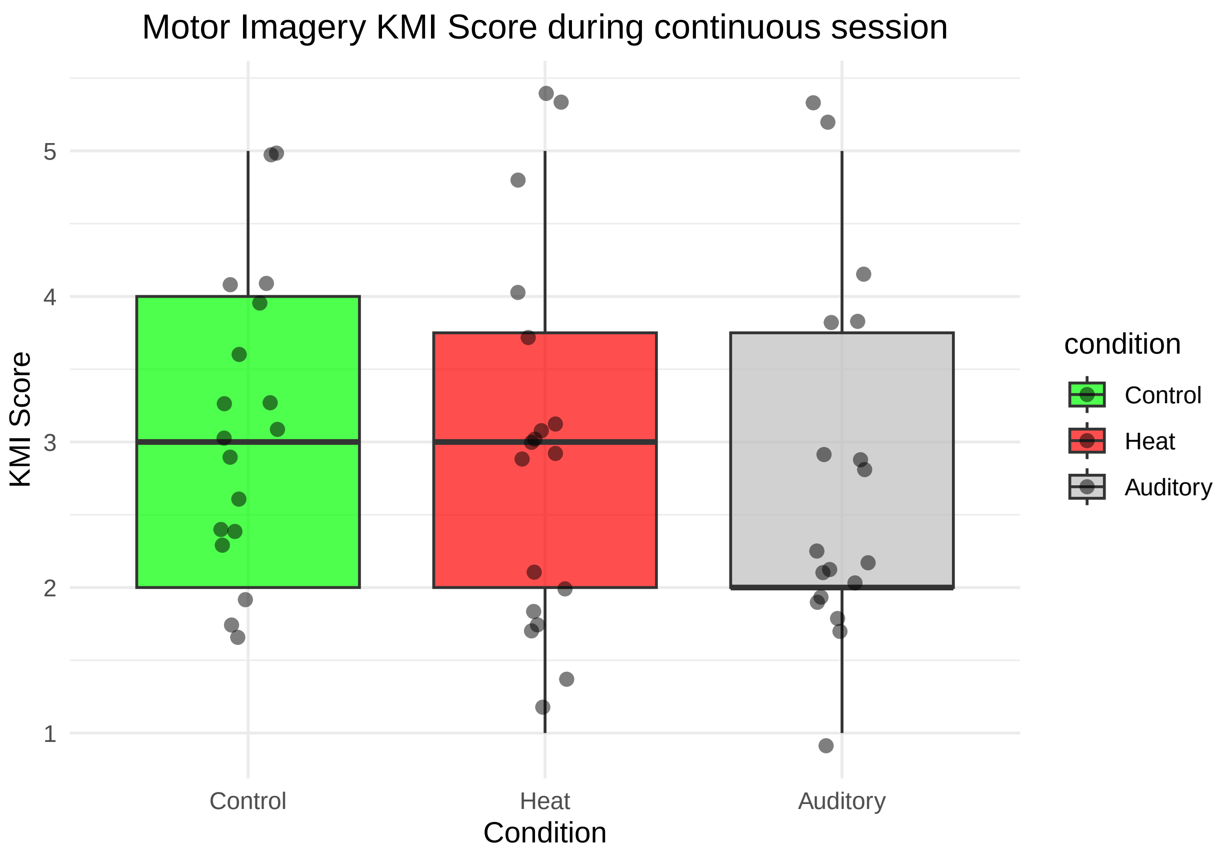

For the VMI score

```
ContinuMI_VMI$condition <- factor(ContinuMI_VMI$condition, levels = c("Control", "Heat", "Auditory"))

compare_means(ContinuMI_VMI ~ condition, data = ContinuMI_VMI)
```

```
## # A tibble: 3 x 8
##   .y.           group1  group2        p p.adj p.format p.signif method
##   <chr>         <chr>   <chr>     <dbl> <dbl> <chr>    <chr>    <chr>
## 1 ContinuMI_VMI Control Heat      0.794  0.79 0.79     ns       Wilcoxon
## 2 ContinuMI_VMI Control Auditory 0.203  0.61 0.20     ns       Wilcoxon
## 3 ContinuMI_VMI Heat    Auditory 0.253  0.61 0.25     ns       Wilcoxon
```

```
my_comparisons <- list( c("Control", "Heat"), c("Control", "Auditory"), c("Heat", "Auditory") )

ggplot(ContinuMI_VMI, aes(x = condition, y = ContinuMI_VMI, fill = condition)) +
  geom_boxplot(alpha = 0.7) +
  geom_jitter(aes(fill = condition), position = position_jitter(0.1), size = 2, alpha = 0.5) +
  labs(title = "Motor Imagery VMI Score during continuous session", x = "Condition", y = "VMI Score") +
  theme_minimal() +
  theme(plot.title = element_text(hjust = 0.5))+
  scale_fill_manual(values = c("Control" = "green", "Heat" = "red", "Auditory" = "grey"))
```

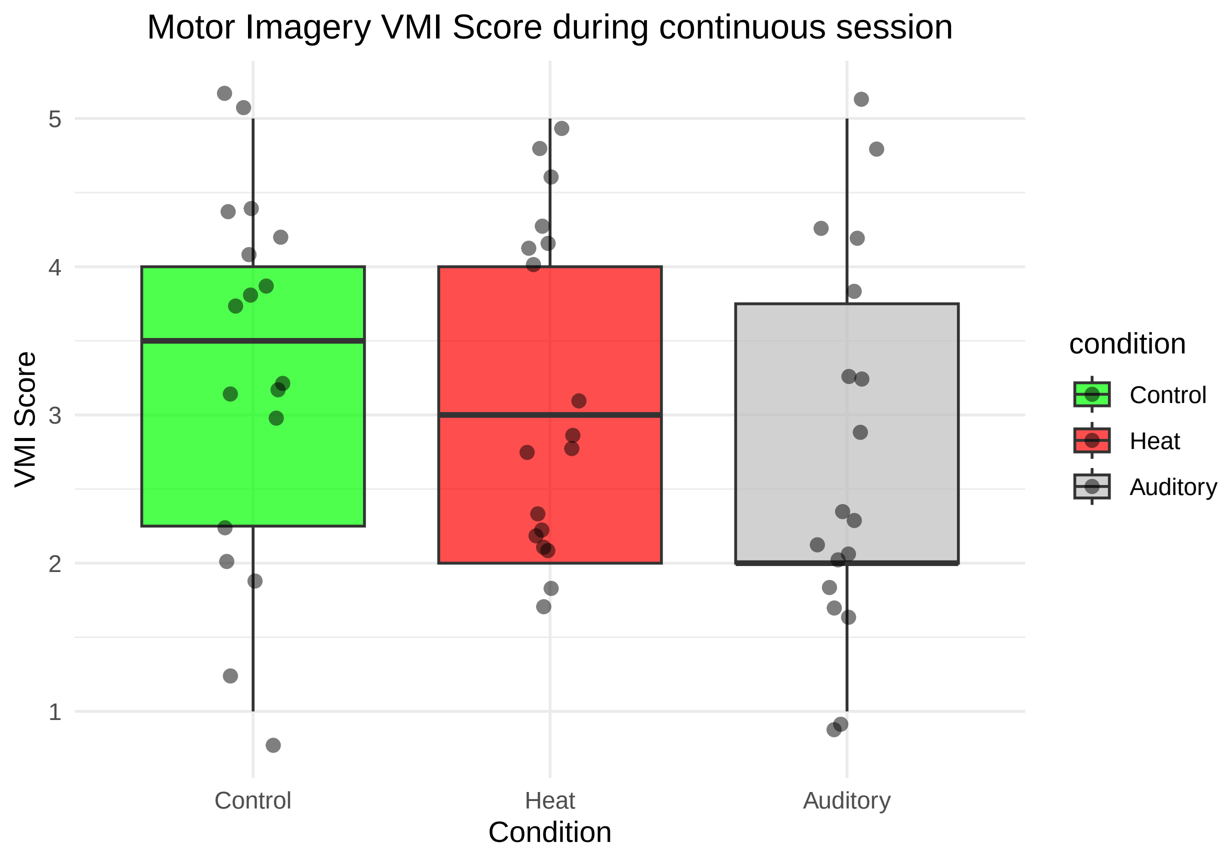

Statistical analysis

```r
friedman_test(ContinuMI_KMI, ContinuMI_KMI ~ condition | ID)
```

For the KMI subscore

```
## # A tibble: 1 x 6
##   .y.               n statistic    df     p method
## * <chr>         <int>     <dbl> <dbl> <dbl> <chr>
## 1 ContinuMI_KMI    18     0.821     2 0.663 Friedman test
```

The friedman test show a non statistically significant effect of condition on KMI Score ($p = 0.663$).

```r
friedman_test(ContinuMI_VMI, ContinuMI_VMI ~ condition | ID)
```

For the VMI subscore

```
## # A tibble: 1 x 6
##   .y.               n statistic    df     p method
## * <chr>         <int>     <dbl> <dbl> <dbl> <chr>
## 1 ContinuMI_VMI    18      4.54     2 0.103 Friedman test
```

The friedman test show a non statistically significant effect of condition on KMI Score (p = 0.103).

```
ContinuMI_KMI %>%
  group_by(condition) %>%
  summarise(
    median = median(ContinuMI_KMI, na.rm = TRUE),
    IQR = IQR(ContinuMI_KMI, na.rm = TRUE),
    CI_low = quantile(ContinuMI_KMI, probs = 0.25, na.rm = TRUE),
    CI_high = quantile(ContinuMI_KMI, probs = 0.75, na.rm = TRUE),
    n = n()
  )
```

Summary of KMI subscore

```
## # A tibble: 3 x 6
##   condition median   IQR CI_low CI_high     n
##   <fct>      <dbl> <dbl>  <dbl>   <dbl> <int>
## 1 Control        3  2         2    4       18
## 2 Heat           3  1.75      2    3.75    18
## 3 Auditory       2  1.75      2    3.75    18
```

```
ContinuMI_VMI %>%
  group_by(condition) %>%
  summarise(
    median = median(ContinuMI_VMI, na.rm = TRUE),
    IQR = IQR(ContinuMI_VMI, na.rm = TRUE),
    CI_low = quantile(ContinuMI_VMI, probs = 0.25, na.rm = TRUE),
    CI_high = quantile(ContinuMI_VMI, probs = 0.75, na.rm = TRUE),
    n = n()
  )
```

Summary of VMI subscore

```
## # A tibble: 3 x 6
##   condition median   IQR CI_low CI_high     n
##   <fct>      <dbl> <dbl>  <dbl>   <dbl> <int>
## 1 Control      3.5  1.75   2.25    4       18
## 2 Heat         3    2      2       4       18
## 3 Auditory     2    1.75   2       3.75    18
```

Motor Imagery Task in the discrete session

```
# Create a dataframe
VAS_MI <- data %>%
  select(ID, Control_IM_VAS, Warm_IM_VAS, Sound_IM_VAS) %>%
```

```r
  pivot_longer(
    cols = c(Control_IM_VAS, Warm_IM_VAS, Sound_IM_VAS),
    names_to = "condition",
    values_to = "VAS_MI"
  ) %>%
  mutate(condition = case_when(
    condition == "Control_IM_VAS" ~ "Control",
    condition == "Warm_IM_VAS" ~ "Heat",
    condition == "Sound_IM_VAS" ~ "Auditory"
  ))

# Detect outliers
na_VAS_MI <- na.omit(VAS_MI$VAS_MI)
outlier(na_VAS_MI)
```

```
## [[1]]
## numeric(0)
##
## [[2]]
## numeric(0)
```

```r
VAS_MI$condition <- factor(VAS_MI$condition, levels = c("Control", "Heat", "Auditory"))

compare_means(VAS_MI ~ condition, data = VAS_MI)
```

Visualize the data

```
## # A tibble: 3 x 8
##   .y.    group1  group2              p      p.adj p.format p.signif method
##   <chr>  <chr>   <chr>           <dbl>      <dbl> <chr>    <chr>    <chr>
## 1 VAS_MI Control Heat     0.0000281   0.000056   2.8e-05  ****     Wilcoxon
## 2 VAS_MI Control Auditory 0.000000293 0.00000088 2.9e-07  ****     Wilcoxon
## 3 VAS_MI Heat    Auditory 0.0184      0.018      0.018    *        Wilcoxon
```

```r
ggplot(VAS_MI, aes(x = condition, y = VAS_MI, fill = condition)) +
  geom_boxplot(alpha = 0.4) +
  geom_jitter(aes(color = condition), position = position_jitter(0.1), size = 2, alpha = 0.8) +
  labs(title = "VAS score during discrete motor imagery", x = "Condition", y = "VAS Score") +
  theme_minimal() +
  theme(plot.title = element_text(hjust = 0.5))+
  scale_fill_manual(values = c("Control" = "green", "Heat" = "red", "Auditory" = "grey"))+
  scale_color_manual(values = c("Control" = "green", "Heat" = "red", "Auditory" = "grey"))
```

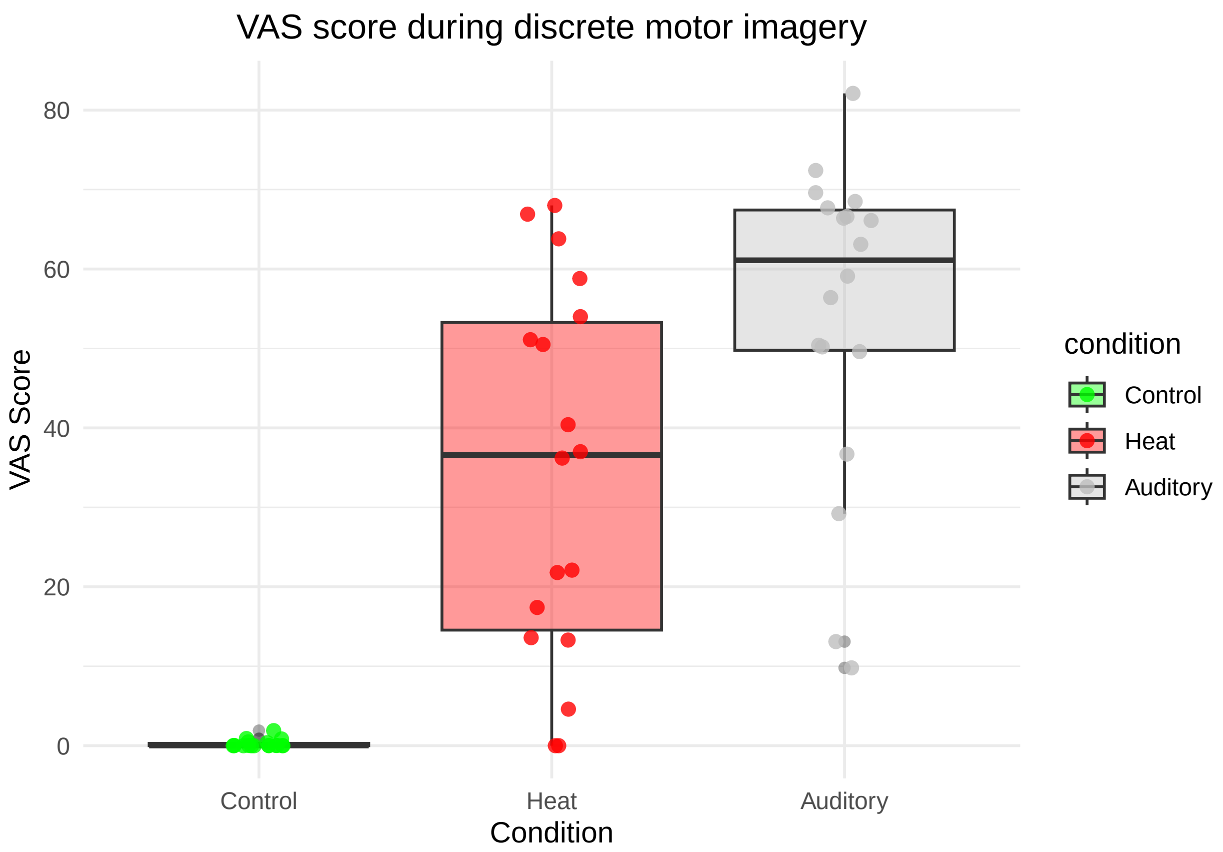

Statistical analysis

```
friedman_test(VAS_MI, VAS_MI ~ condition | ID)
```

```
## # A tibble: 1 x 6
##   .y.        n statistic    df          p method
## * <chr>  <int>     <dbl> <dbl>      <dbl> <chr>
## 1 VAS_MI    18      25.3     2 0.00000315 Friedman test
```

The friedman test show a statistically significant effect of condition on VAS during MI task ($p < 0.001$).

```
VAS_MI %>% wilcox_test(VAS_MI ~ condition, paired = TRUE, p.adjust.method = "bonferroni")
```

```
## # A tibble: 3 x 9
##   .y.    group1  group2      n1    n2 statistic          p    p.adj p.adj.signif
## * <chr>  <chr>   <chr>    <int> <int>     <dbl>      <dbl>    <dbl> <chr>
## 1 VAS_MI Control Heat        18    18         3 0.000355    1    e-3 **
## 2 VAS_MI Control Auditory    18    18         0 0.00000763  2.29e-5 ****
## 3 VAS_MI Heat    Auditory    18    18        20 0.003       8    e-3 **
```

```
VAS_MI %>% wilcox_effsize(VAS_MI ~ condition, paired = TRUE)
```

```
## # A tibble: 3 x 7
##   .y.    group1  group2   effsize    n1    n2 magnitude
## * <chr>  <chr>   <chr>      <dbl> <int> <int> <ord>
## 1 VAS_MI Control Heat       0.847    18    18 large
## 2 VAS_MI Control Auditory   0.878    18    18 large
## 3 VAS_MI Heat    Auditory   0.672    18    18 large
```

Post hoc shows a significant difference between the Control and Auditory conditions ($p < 0.001$) with a large effect size (0.878), between the Auditory and Heat conditions ($p < 0.001$) with a moderate effect size (0.672), and between the Control and Heat conditions ($p < 0.001$) with a large effect size (0.796).

Summary of VAS score during Motor Imagery

```
VAS_MI %>%
  group_by(condition) %>%
  summarise(
    mean = mean(VAS_MI, na.rm = TRUE),
    sd = sd(VAS_MI, na.rm = TRUE),
    n = n(),
    # get the standard error of the mean
    sem = sd / sqrt(n),
    # get the 95% confidence interval
    ymin = mean - 1.96 * sem,
    ymax = mean + 1.96 * sem
  )
```

```
## # A tibble: 3 x 7
##   condition   mean    sd     n   sem    ymin   ymax
##   <fct>      <dbl> <dbl> <int> <dbl>   <dbl>  <dbl>
## 1 Control    0.261 0.503    18 0.119  0.0282  0.493
## 2 Heat      34.4   23.4     18 5.53  23.6    45.2
## 3 Auditory  54.3   20.2     18 4.76  45.0    63.6
```

Conclusion

The results of the analysis indicate that the unpleasant stimuli highly increase the VAS intensity, in addition the auditory unpleasant stimulus is more unpleasant than the heat condition during MI tasks.

VAS during motor execution in the discrete session

```
# Create a dataframe
VAS_Exe <- data %>%
  select(ID, Control_Exe_VAS, Warm_Exe_VAS, Sound_Exe_VAS) %>%
  pivot_longer(
    cols = c(Control_Exe_VAS, Warm_Exe_VAS, Sound_Exe_VAS),
    names_to = "condition",
    values_to = "VAS_Exe"
  ) %>%
  mutate(condition = case_when(
```

```r
    condition == "Control_Exe_VAS" ~ "Control",
    condition == "Warm_Exe_VAS" ~ "Heat",
    condition == "Sound_Exe_VAS" ~ "Auditory"
  ))

# Detect outliers
na_VAS_Exe<- na.omit(VAS_Exe$VAS_Exe)
outlier(na_VAS_Exe)
```

```
## [[1]]
## numeric(0)
##
## [[2]]
## numeric(0)
```

Visualize the data

```r
VAS_Exe$condition <- factor(VAS_Exe$condition, levels = c("Control", "Heat", "Auditory"))

ggplot(VAS_Exe, aes(x = condition, y = VAS_Exe, fill = condition)) +
  geom_boxplot(alpha = 0.4) +
  geom_jitter(aes(color = condition), position = position_jitter(0.1), size = 2, alpha = 0.8) +
  labs(title = "VAS score during discrete motor execution", x = "Condition", y = "VAS Score") +
  theme_minimal() +
  theme(plot.title = element_text(hjust = 0.5))+
  scale_fill_manual(values = c("Control" = "green", "Heat" = "red", "Auditory" = "grey"))+
  scale_color_manual(values = c("Control" = "green", "Heat" = "red", "Auditory" = "grey"))
```

```
## Warning: Removed 8 rows containing non-finite outside the scale range
## (`stat_boxplot()`).
```

```
## Warning: Removed 8 rows containing missing values or values outside the scale range
## (`geom_point()`).
```

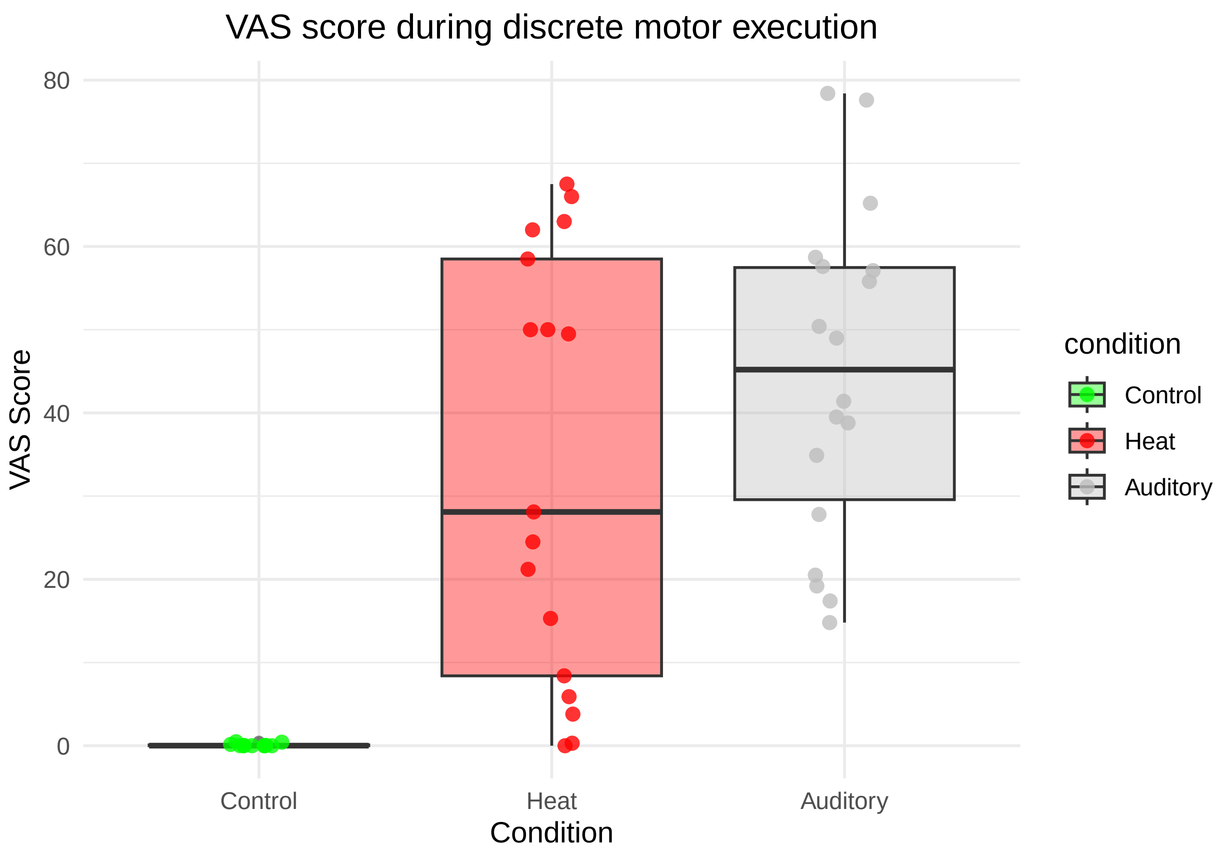

Statistical analysis

```
# Checking for missing values
colSums(is.na(VAS_Exe))
```

```
##        ID condition   VAS_Exe
##         0         0         8
```

```
# count the number of missing values for each participant
VAS_Exe %>%
  group_by(ID) %>%
  summarise(n_NA = sum(is.na(VAS_Exe)), n_total = n()) %>%
  filter(n_NA >= (n_total - 1))
```

```
## # A tibble: 1 x 3
##       ID  n_NA n_total
##    <int> <int>   <int>
## 1     1     2       3
```

```
# delete the participant with missing values
VAS_Exe_clean <- VAS_Exe %>%
  group_by(ID) %>%
  filter(sum(!is.na(VAS_Exe)) > 1) %>%
  ungroup()
```

Due to 8 missing values, another non parametric test will be used for the analysis such as Skillings Mack.test

```
Ski.Mack(y = VAS_Exe_clean$VAS_Exe,
                    groups = VAS_Exe_clean$condition,
                    blocks = VAS_Exe_clean$ID)
```

```
##
## Skillings-Mack Statistic =  17.231256 , p-value =  0.000181
## Note: the p-value is based on the chi-squared distribution with d.f. =  2
##
## $Nblocks
## [1] 17
##
## $Ntreatments
## [1] 3
##
## $rawdata
##      [,1] [,2]  [,3]  [,4] [,5] [,6]  [,7]  [,8] [,9] [,10] [,11] [,12] [,13]
## [1,]   NA  0.0  0.48  0.04   NA   NA  0.02  0.42  0.0    NA   0.0    NA    NA
## [2,] 28.1 50.0 21.20 49.50 67.5  0.3 63.00 66.00 62.0   8.4  50.0  15.3  24.5
## [3,] 58.7 17.4 38.80 77.60 49.0 39.5 57.60 50.40 65.2  78.4  57.1  55.8  27.8
##      [,14] [,15] [,16] [,17]
## [1,]   0.0   0.0  0.01  0.16
## [2,]   0.0   5.9  3.80 58.50
## [3,]  20.5  14.8 19.20 34.90
##
## $rankdata
##      [,1] [,2] [,3] [,4] [,5] [,6] [,7] [,8] [,9] [,10] [,11] [,12] [,13] [,14]
## [1,]  1.5    1    1    1  1.5  1.5    1    1    1   1.5     1   1.5   1.5   1.5
## [2,]  1.0    3    2    2  2.0  1.0    3    3    2   1.0     2   1.0   1.0   1.5
## [3,]  2.0    2    3    3  1.0  2.0    2    2    3   2.0     3   2.0   2.0   3.0
##      [,15] [,16] [,17]
## [1,]     1     1     1
## [2,]     2     2     3
## [3,]     3     3     2
##
## $varCovarMatrix
##      [,1] [,2] [,3]
## [1,]   22  -11  -11
## [2,]  -11   28  -17
## [3,]  -11  -17   28
##
## $adjustedSum
##              [,1]        [,2]        [,3]
## [1,] -18.18653348 2.062177826 16.12435565
```

The Skilling Mack test show a statistically significant effect of condition on VAS during motor execution ($p < 0.001$).

```
#Post hoc analysis
wilcox_test(VAS_Exe, VAS_Exe ~ condition, paired = TRUE, p.adjust.method = "bonferroni")
```

```
## # A tibble: 3 x 9
```

```
##   .y.     group1  group2      n1    n2 statistic        p p.adj p.adj.signif
## * <chr>   <chr>   <chr>    <int> <int>     <dbl>    <dbl> <dbl> <chr>
## 1 VAS_Exe Control Heat        11    17         0 0.006    0.018 *
## 2 VAS_Exe Control Auditory    11    18         0 0.000977 0.003 **
## 3 VAS_Exe Heat    Auditory    17    18        44 0.132    0.396 ns
```

```
#Effect size
wilcox_effsize(VAS_Exe ~ condition, data = VAS_Exe, paired = TRUE)
```

```
## # A tibble: 3 x 7
##   .y.     group1  group2   effsize    n1    n2 magnitude
## * <chr>   <chr>   <chr>      <dbl> <int> <int> <ord>
## 1 VAS_Exe Control Heat       0.873    11    17 large
## 2 VAS_Exe Control Auditory   0.885    11    18 large
## 3 VAS_Exe Heat    Auditory   0.373    17    18 moderate
```

Post hoc shows a significant difference between the Control and Auditory conditions ($p < 0.001$) with a large effect size (1.855), and between the Control and Heat conditions ($p = 0.002$) with a large effect size (1.476).

Summary of VAS score during Motor Execution

```
VAS_Exe %>%
  group_by(condition) %>%
  summarise(
    mean = mean(VAS_Exe, na.rm = TRUE),
    sd = sd(VAS_Exe, na.rm = TRUE),
    n = n(),
    # get the standard error of the mean
    sem = sd / sqrt(n),
    # get the 95% confidence interval
    ymin = mean - 1.96 * sem,
    ymax = mean + 1.96 * sem
  )
```

```
## # A tibble: 3 x 7
##   condition   mean     sd     n    sem    ymin   ymax
##   <fct>      <dbl>  <dbl> <int>  <dbl>   <dbl>  <dbl>
## 1 Control    0.103  0.178    18 0.0421  0.0203  0.185
## 2 Heat      33.8   25.5      18 6.01   22.0    45.5
## 3 Auditory  44.7   19.8      18 4.66   35.5    53.8
```

Conclusion

The results of the analysis indicate that the unpleasant stimuli highly increase the VAS intensity during ME tasks.

VAS during motor imagery in the continuous session

```r
# Transform columns as numeric
data$ContinuWarm_IM_VAS <- as.numeric(data$ContinuWarm_IM_VAS)
data$ContinuSound_IM_VAS <- as.numeric(data$ContinuSound_IM_VAS)

# Create a dataframe
ContinuVAS_MI <- data %>%
  select(ID, Control_IM_VAS, ContinuWarm_IM_VAS, ContinuSound_IM_VAS) %>%
  pivot_longer(
    cols = c(Control_IM_VAS, ContinuWarm_IM_VAS, ContinuSound_IM_VAS),
    names_to = "condition",
    values_to = "VAS_MI"
  ) %>%
  mutate(condition = case_when(
    condition == "Control_IM_VAS" ~ "Control",
    condition == "ContinuWarm_IM_VAS" ~ "Heat",
    condition == "ContinuSound_IM_VAS" ~ "Auditory"
  ))

# Detect outliers
na_ContinuVAS_MI<- na.omit(ContinuVAS_MI$VAS_MI)
outlier(na_ContinuVAS_MI)
```

```
## [[1]]
## numeric(0)
##
## [[2]]
## numeric(0)
```

Visualize the data

```r
ContinuVAS_MI$condition <- factor(ContinuVAS_MI$condition, levels = c("Control", "Heat", "Auditory"))

ggplot(ContinuVAS_MI, aes(x = condition, y = VAS_MI, fill = condition)) +
  geom_boxplot(alpha = 0.4) +
  geom_jitter(aes(color = condition), position = position_jitter(0.1), size = 2, alpha = 0.8) +
  labs(title = "VAS score during continuous motor imagery", x = "Condition", y = "VAS Score") +
  theme_minimal() +
  theme(plot.title = element_text(hjust = 0.5))+
  scale_fill_manual(values = c("Control" = "green", "Heat" = "red", "Auditory" = "grey"))+
  scale_color_manual(values = c("Control" = "green", "Heat" = "red", "Auditory" = "grey"))
```

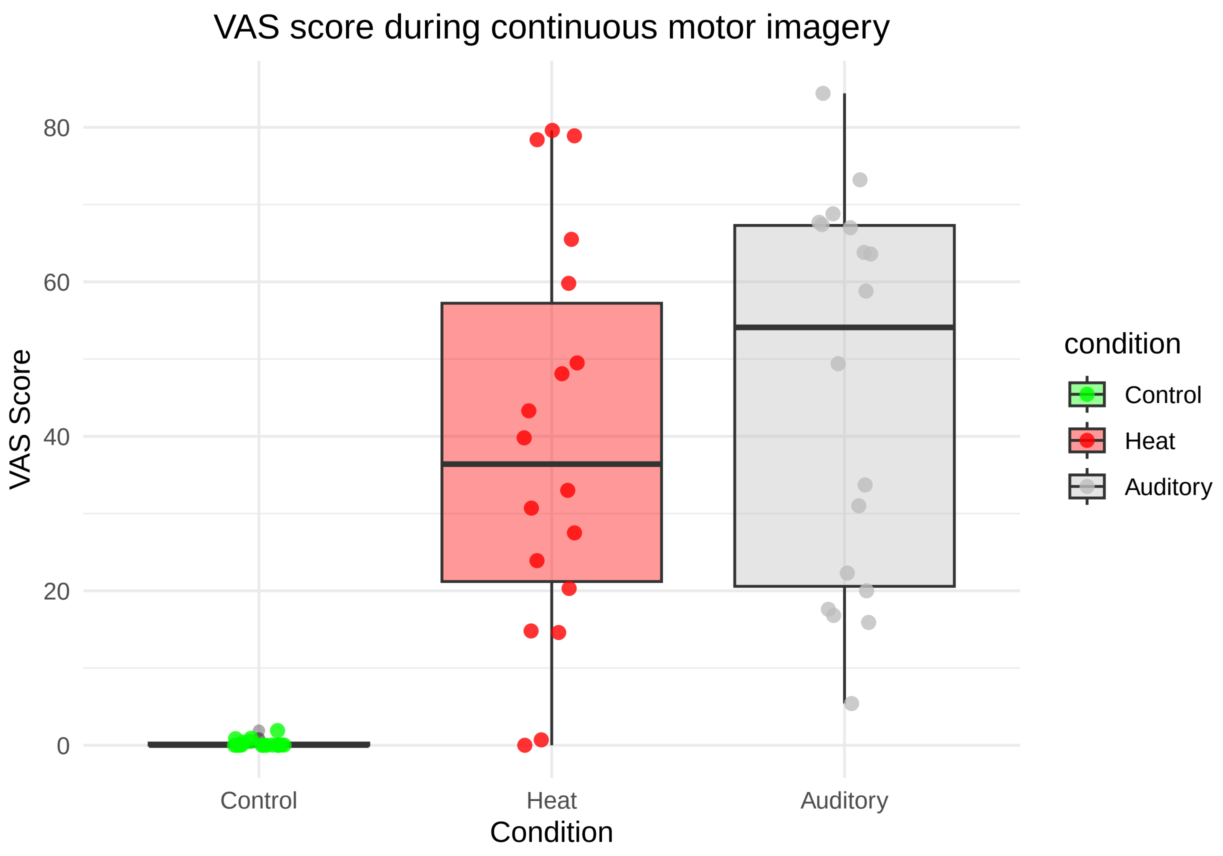

Statistical analysis

```r
friedman_test(ContinuVAS_MI, VAS_MI ~ condition | ID)
```

```
## # A tibble: 1 x 6
##   .y.        n statistic    df         p method
## * <chr>  <int>     <dbl> <dbl>     <dbl> <chr>
## 1 VAS_MI    18      21.4     2 0.0000220 Friedman test
```

The friedman test show a statistically significant effect of condition on VAS during MI task ($p < 0.001$).

```r
#Post hoc analysis
wilcox_test(ContinuVAS_MI, VAS_MI ~ condition, paired = TRUE, p.adjust.method = "bonferroni")
```

```
## # A tibble: 3 x 9
##   .y.    group1  group2      n1    n2 statistic          p    p.adj p.adj.signif
## * <chr>  <chr>   <chr>    <int> <int>     <dbl>      <dbl>    <dbl> <chr>
## 1 VAS_MI Control Heat        18    18         3 0.0000381   1.14e-4 ***
## 2 VAS_MI Control Auditory    18    18         0 0.00000763  2.29e-5 ****
## 3 VAS_MI Heat    Auditory    18    18        67 0.442       1   e+0 ns
```

```r
#Effect size
wilcox_effsize(VAS_MI ~ condition, data = ContinuVAS_MI, paired = TRUE)
```

```
## # A tibble: 3 x 7
##   .y.    group1  group2   effsize    n1    n2 magnitude
## * <chr>  <chr>   <chr>      <dbl> <int> <int> <ord>
## 1 VAS_MI Control Heat       0.847    18    18 large
## 2 VAS_MI Control Auditory   0.878    18    18 large
## 3 VAS_MI Heat    Auditory   0.190    18    18 small
```

Post hoc shows a significant difference between the Control and Auditory conditions ($p < 0.001$) with a large effect size (1.634), between the Auditory and Heat conditions ($p < 0.001$) with a small effect size (0.25), and between the Control and Heat conditions ($p < 0.001$) with a large effect size (1.34).

Summary of VAS score during Motor Imagery

```r
ContinuVAS_MI %>%
  group_by(condition) %>%
  summarise(
    mean = mean(VAS_MI, na.rm = TRUE),
    sd = sd(VAS_MI, na.rm = TRUE),
    n = n(),
    # get the standard error of the mean
    sem = sd / sqrt(n),
    # get the 95% confidence interval
    ymin = mean - 1.96 * sem,
    ymax = mean + 1.96 * sem
  )
```

```
## # A tibble: 3 x 7
##   condition   mean     sd     n   sem    ymin   ymax
##   <fct>      <dbl>  <dbl> <int> <dbl>   <dbl>  <dbl>
## 1 Control    0.261  0.503    18 0.119  0.0282  0.493
## 2 Heat      39.4   25.6      18 6.03  27.5    51.2
## 3 Auditory  45.9   25.1      18 5.92  34.3    57.5
```

Conclusion

The results of the analysis indicate that the unpleasant stimuli significantly increase the VAS intensity. Additionally, the auditory unpleasant stimulus is perceived as more unpleasant than the heat condition during continuous MI tasks, although the difference between the auditory and heat conditions has a small effect.

VAS during Motor execution during continuous session

```r
# Transform columns as numeric
data$ContinuWarm_Exe_VAS <- as.numeric(data$ContinuWarm_Exe_VAS)
data$ContinuSound_Exe_VAS <- as.numeric(data$ContinuSound_Exe_VAS)
```

```r
# Create a dataframe
ContinuVAS_Exe <- data %>%
  select(ID, Control_Exe_VAS, ContinuWarm_Exe_VAS, ContinuSound_Exe_VAS) %>%
  pivot_longer(
    cols = c(Control_Exe_VAS, ContinuWarm_Exe_VAS, ContinuSound_Exe_VAS),
    names_to = "condition",
    values_to = "VAS_Exe"
  ) %>%
  mutate(condition = case_when(
    condition == "Control_Exe_VAS" ~ "Control",
    condition == "ContinuWarm_Exe_VAS" ~ "Heat",
    condition == "ContinuSound_Exe_VAS" ~ "Auditory"
  ))

# Detect outliers
na_ContinuVAS_Exe<- na.omit(ContinuVAS_Exe$VAS_Exe)
outlier(na_ContinuVAS_Exe)
```

```
## [[1]]
## numeric(0)
##
## [[2]]
## numeric(0)
```

Visualize the data

```r
ContinuVAS_Exe$condition <- factor(ContinuVAS_Exe$condition, levels = c("Control", "Heat", "Auditory"))

ggplot(ContinuVAS_Exe, aes(x = condition, y = VAS_Exe, fill = condition)) +
  geom_boxplot(alpha = 0.4) +
  geom_jitter(aes(color = condition), position = position_jitter(0.1), size = 2, alpha = 0.8) +
  labs(title = "VAS score during continuous motor execution", x = "Condition", y = "VAS Score") +
  theme_minimal() +
  theme(plot.title = element_text(hjust = 0.5))+
  scale_fill_manual(values = c("Control" = "green", "Heat" = "red", "Auditory" = "grey"))+
  scale_color_manual(values = c("Control" = "green", "Heat" = "red", "Auditory" = "grey"))
```

```
## Warning: Removed 7 rows containing non-finite outside the scale range
## (`stat_boxplot()`).

## Warning: Removed 7 rows containing missing values or values outside the scale range
## (`geom_point()`).
```

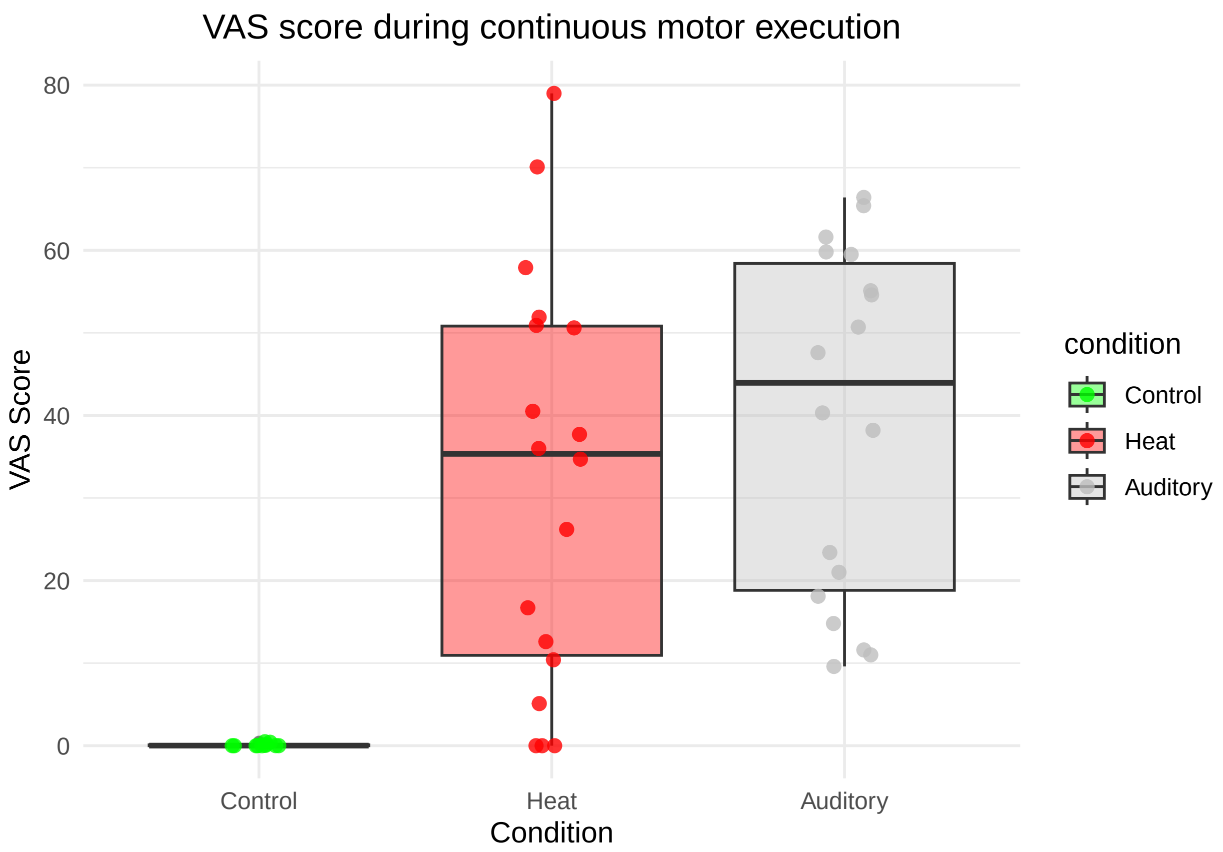

Statistical analysis

```
# Checking for missing values
colSums(is.na(ContinuVAS_Exe))
```

```
##        ID condition   VAS_Exe
##         0         0         7
```

```
# count the number of missing values for each participant
ContinuVAS_Exe %>%
  group_by(ID) %>%
  summarise(n_NA = sum(is.na(ContinuVAS_Exe)), n_total = n()) %>%
  filter(n_NA >= (n_total - 1))
```

```
## # A tibble: 18 x 3
##       ID  n_NA n_total
##    <int> <int>   <int>
##  1     1     7       3
##  2     2     7       3
##  3     3     7       3
##  4     4     7       3
##  5     5     7       3
##  6     6     7       3
##  7     7     7       3
##  8     8     7       3
##  9     9     7       3
```

```
## 10    10     7       3
## 11    11     7       3
## 12    12     7       3
## 13    13     7       3
## 14    14     7       3
## 15    15     7       3
## 16    16     7       3
## 17    17     7       3
## 18    18     7       3
```

```
# delete the participant with missing values
ContinuVAS_Exe_clean <- ContinuVAS_Exe %>%
  group_by(ID) %>%
  filter(sum(!is.na(ContinuVAS_Exe)) > 1) %>%
  ungroup()
```

Due to 7 missing values, another non parametric test will be used for the analysis such as Skillings Mack.test

```
Ski.Mack(y = ContinuVAS_Exe_clean$VAS_Exe,
                    groups = ContinuVAS_Exe_clean$condition,
                    blocks = ContinuVAS_Exe_clean$ID)
```

```
##
## Skillings-Mack Statistic =  14.331081 , p-value =  0.000773
## Note: the p-value is based on the chi-squared distribution with d.f. =  2
##
## $Nblocks
## [1] 18
##
## $Ntreatments
## [1] 3
##
## $rawdata
##      [,1] [,2] [,3]  [,4]  [,5] [,6] [,7]  [,8]  [,9] [,10] [,11] [,12] [,13]
## [1,]   NA   NA  0.0  0.48  0.04   NA   NA  0.02  0.42   0.0    NA   0.0    NA
## [2,] 34.7 10.4 26.2 37.70 51.90 79.0 50.9 57.90 40.50  70.1  16.7  36.0   0.0
## [3,] 38.2 54.6 47.6 18.10 61.60 55.1 40.3 65.40 23.40  66.4  59.8  14.8  59.5
##      [,14] [,15] [,16] [,17] [,18]
## [1,]    NA   0.0   0.0  0.01  0.16
## [2,]  12.6   0.0   5.1  0.00 50.60
## [3,]  21.0  11.6   9.6 11.00 50.70
##
## $rankdata
##      [,1] [,2] [,3] [,4] [,5] [,6] [,7] [,8] [,9] [,10] [,11] [,12] [,13] [,14]
## [1,]  1.5  1.5    1    1    1  1.5  1.5    1    1     1   1.5     1   1.5   1.5
## [2,]  1.0  1.0    2    3    2  2.0  2.0    2    3     3   1.0     3   1.0   1.0
## [3,]  2.0  2.0    3    2    3  1.0  1.0    3    2     2   2.0     2   2.0   2.0
##      [,15] [,16] [,17] [,18]
## [1,]   1.5     1     2     1
## [2,]   1.5     2     1     2
## [3,]   3.0     3     3     3
##
## $varCovarMatrix
```

```
##      [,1] [,2] [,3]
## [1,]   22  -11  -11
## [2,]  -11   29  -18
## [3,]  -11  -18   29
##
## $adjustedSum
##              [,1]        [,2]        [,3]
## [1,] -16.45448267 1.330127019 15.12435565
```

The Skilling mack test show a statistically significant effect of condition on VAS during motor execution ($p < 0.001$).

```r
#Post hoc analysis
wilcox_test(ContinuVAS_Exe, VAS_Exe ~ condition, paired = TRUE, p.adjust.method = "bonferroni")
```

```
## # A tibble: 3 x 9
##   .y.     group1  group2      n1    n2 statistic        p p.adj p.adj.signif
## * <chr>   <chr>   <chr>    <int> <int>     <dbl>    <dbl> <dbl> <chr>
## 1 VAS_Exe Control Heat        11    18         1 0.008    0.024 *
## 2 VAS_Exe Control Auditory    11    18         0 0.000977 0.003 **
## 3 VAS_Exe Heat    Auditory    18    18        62 0.325    0.975 ns
```

```r
#Effect size
wilcox_effsize(VAS_Exe ~ condition, data = ContinuVAS_Exe, paired = TRUE)
```

```
## # A tibble: 3 x 7
##   .y.     group1  group2   effsize    n1    n2 magnitude
## * <chr>   <chr>   <chr>      <dbl> <int> <int> <ord>
## 1 VAS_Exe Control Heat       0.818    11    18 large
## 2 VAS_Exe Control Auditory   0.885    11    18 large
## 3 VAS_Exe Heat    Auditory   0.241    18    18 small
```

Post hoc shows a significant difference between the Control and Auditory conditions ($p < 0.001$) with a large effect size (1.390) and between the Control and Heat conditions ($p < 0.001$) with a large effect size (1.3993).

Summary of VAS score during Motor Execution

```r
ContinuVAS_Exe %>%
  group_by(condition) %>%
  summarise(
    mean = mean(VAS_Exe, na.rm = TRUE),
    sd = sd(VAS_Exe, na.rm = TRUE),
    n = n(),
    # get the standard error of the mean
    sem = sd / sqrt(n),
    # get the 95% confidence interval
    ymin = mean - 1.96 * sem,
    ymax = mean + 1.96 * sem
  )
```

```
## # A tibble: 3 x 7
##   condition   mean     sd     n    sem    ymin   ymax
##   <fct>      <dbl>  <dbl> <int>  <dbl>   <dbl>  <dbl>
## 1 Control    0.103  0.178    18 0.0421  0.0203  0.185
## 2 Heat      32.2   24.8      18 5.85   20.8    43.7
## 3 Auditory  39.4   21.0      18 4.96   29.7    49.1
```

Conclusion

The results of the analysis indicate that the unpleasant stimuli significantly increase the VAS intensity.

correlations between VAS and MI abilities

During discrete session

```r
#Select variables
VAS_score <- data %>%
  select(ID, Control_IM_VAS, Warm_IM_VAS, Sound_IM_VAS) %>%
  pivot_longer(
    cols = c(Control_IM_VAS, Warm_IM_VAS, Sound_IM_VAS),
    names_to = "condition",
    values_to = "VAS"
  ) %>%
  mutate(condition = case_when(
    condition == "Control_IM_VAS" ~ "Control",
    condition == "Warm_IM_VAS" ~ "Heat",
    condition == "Sound_IM_VAS" ~ "Auditory"
  ))%>%
  filter(condition != "Control")
```

```r
#Filtering data without Healthy group
Filt_MI_Total <- MI_Total %>%
  filter(condition != "Control")

Filt_MI_KMI <- MI_KMI %>%
  filter(condition != "Control")

Filt_MI_VMI <- MI_VMI %>%
  filter(condition != "Control")

df1_list <- list(MI_Total, VAS_score)
cor_var_Total <-  merge(Filt_MI_Total, VAS_score, by = c("ID", "condition"))
cor_var_KMI <-  merge(Filt_MI_KMI, VAS_score, by = c("ID", "condition"))
cor_var_VMI <-  merge(Filt_MI_VMI, VAS_score, by = c("ID", "condition"))
```

```r
#Plot correlation
gg_cor_Total <- ggplot(cor_var_Total, aes(x=VAS, y=MI_TotalScore, color = condition, shape = condition)
  geom_point() +
```

```
  geom_smooth(method=lm, aes(color = condition, fill = condition))+
  stat_cor(method = "pearson", label.x=6)+
    labs(title = "Correlation between VAS and MI Total score during discrete motion session", y = "MI To
  scale_color_manual(values = c("Auditory" = "grey", "Heat" = "red"))+
  theme_minimal()+
  scale_fill_manual(values = c("Auditory" = "grey", "Heat" = "red"))

gg_cor_KMI <- ggplot(cor_var_KMI, aes(x=VAS, y=MI_KMI, color = condition, shape = condition)) +
  geom_point() +
  geom_smooth(method=lm, aes(color = condition, fill = condition))+
  stat_cor(method = "pearson", label.x=6)+
    labs(title = "Correlation between VAS and KMI score during discrete motion session", y = "KMI Score
  scale_color_manual(values = c("Auditory" = "grey", "Heat" = "red"))+
  theme_minimal()+
  scale_fill_manual(values = c("Auditory" = "grey", "Heat" = "red"))

gg_cor_VMI <- ggplot(cor_var_VMI, aes(x=VAS, y=MI_VMI, color = condition, shape = condition)) +
  geom_point() +
  geom_smooth(method=lm, aes(color = condition, fill = condition))+
  stat_cor(method = "pearson", label.x=6)+
    labs(title = "Correlation between VAS and VMI score during discrete motion session", y = "VMI Score
  scale_color_manual(values = c("Auditory" = "grey", "Heat" = "red"))+
  theme_minimal()+
  scale_fill_manual(values = c("Auditory" = "grey", "Heat" = "red"))


print(gg_cor_Total)
```

Visualize the data

```
## `geom_smooth()` using formula = 'y ~ x'
```

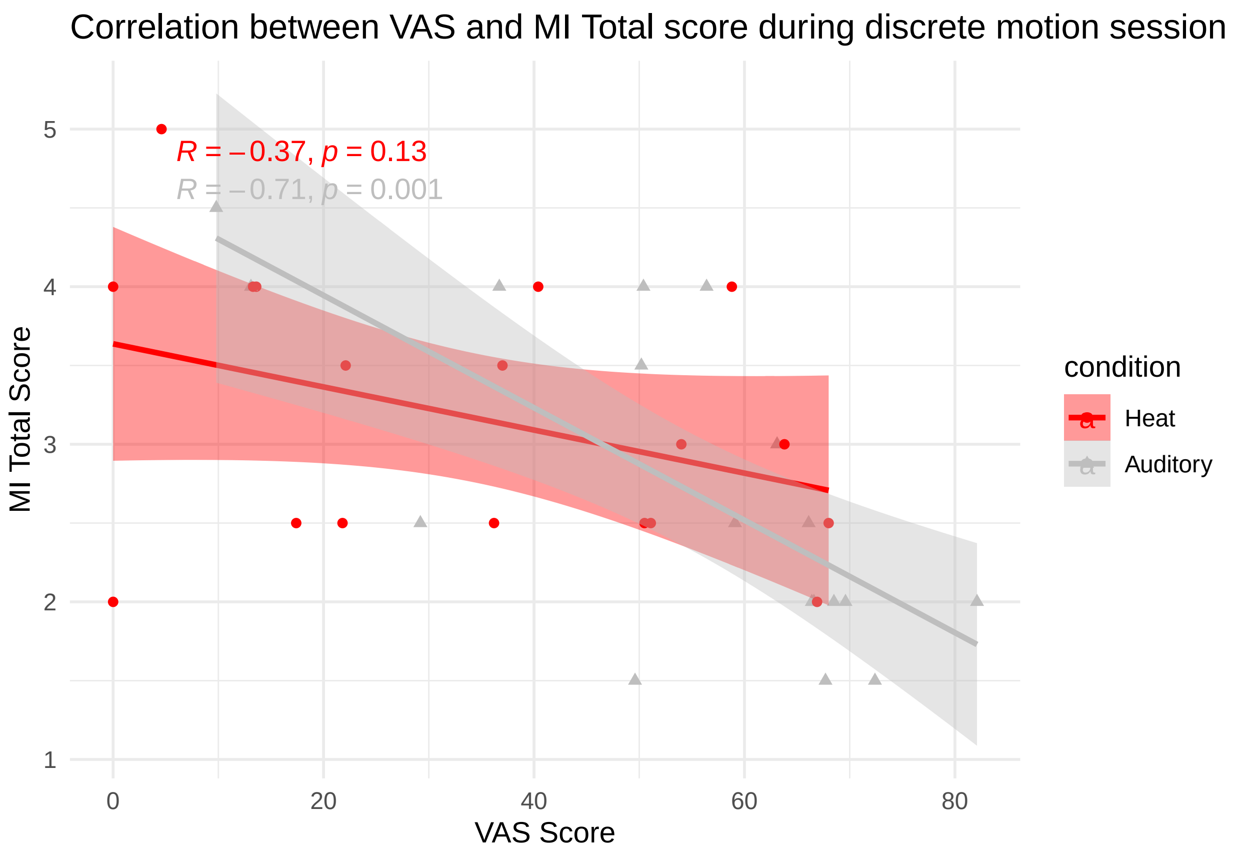


```r
print(gg_cor_KMI)
```

```
## `geom_smooth()` using formula = 'y ~ x'
```

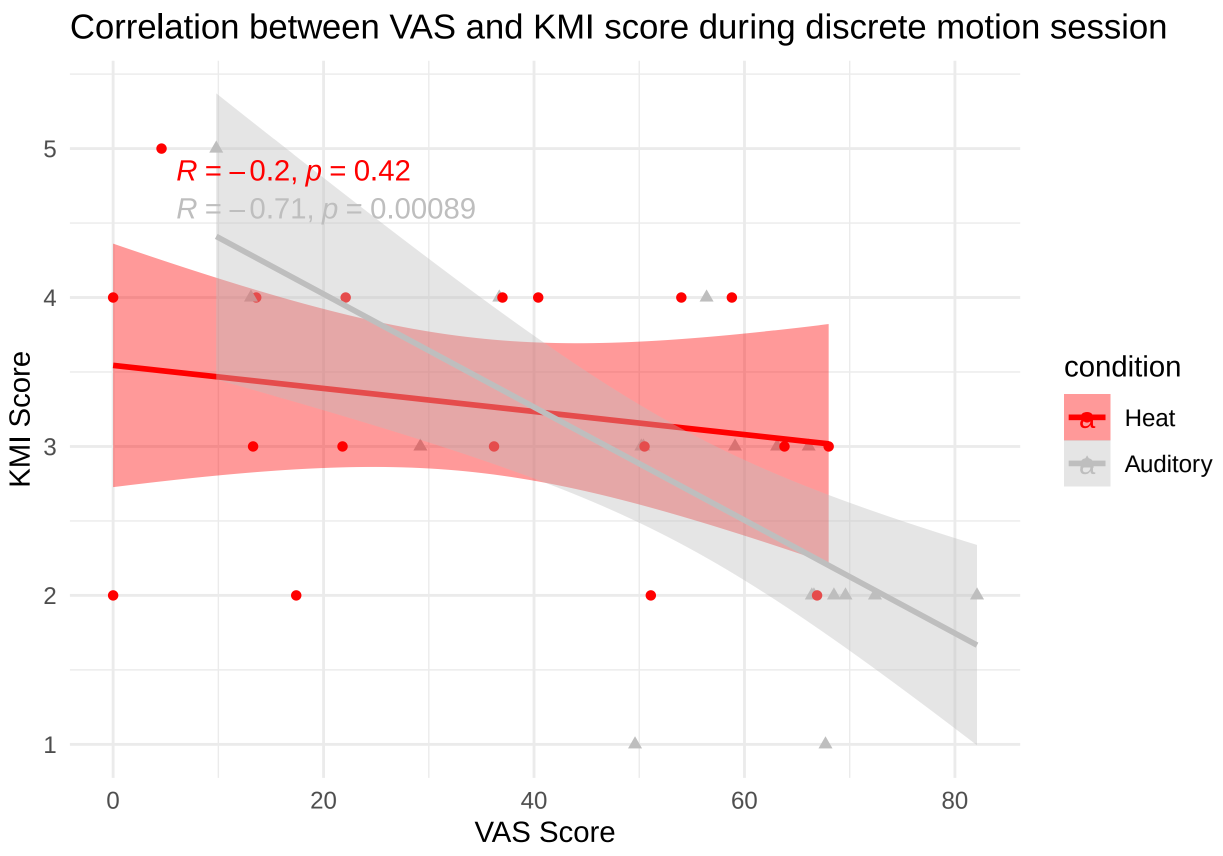


```r
print(gg_cor_VMI)
```

```
## `geom_smooth()` using formula = 'y ~ x'
```

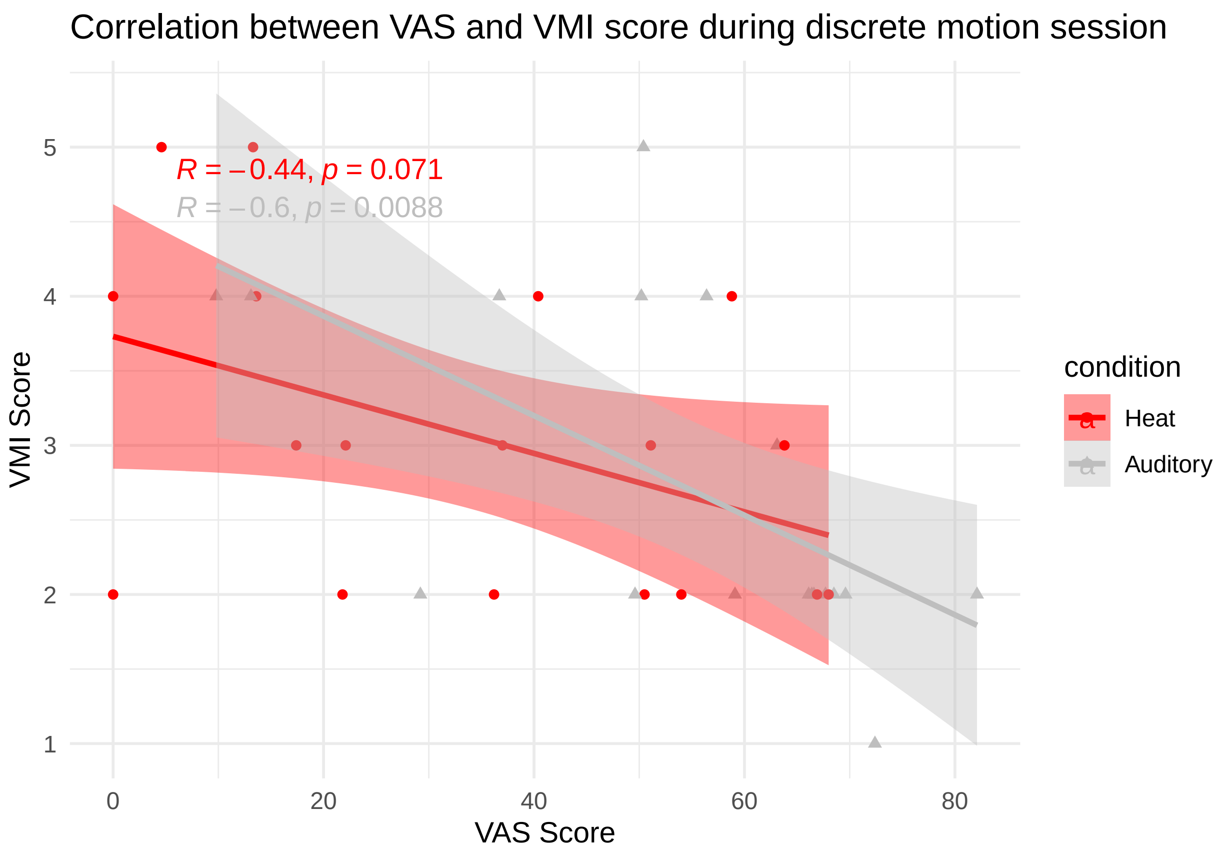

Conclusion

The results of the analysis indicate that there is a significant negative correlation between the VAS score and the MI abilities during discrete motion session, only for the auditory condition. The auditory unpleasant stimulus has a high negative impact on MI abilities compared to the heat and Control conditions.

During Continuous session

```
#Filtering data without Healthy group
Filt_ContinuMI_Total <- ContinuMI_Total %>%
  filter(condition != "Control")

Filt_CContinuMI_KMI <- ContinuMI_KMI %>%
  filter(condition != "Control")

Filt_ContinuMI_VMI1 <- ContinuMI_VMI %>%
  filter(condition != "Control")

cor_var_ContinuTotal <-  merge(ContinuMI_Total, VAS_score, by = c("ID", "condition"))
cor_var_ContinuKMI <-  merge(ContinuMI_KMI, VAS_score, by = c("ID", "condition"))
cor_var_ContinuVMI <-  merge(ContinuMI_VMI, VAS_score, by = c("ID", "condition"))
```

```
#Plot correlation
gg_cor_ContinuTotal <- ggplot(cor_var_ContinuTotal, aes(x=VAS, y=ContinuMI_TotalScore, color = conditio
  geom_point() +
  geom_smooth(method=lm, aes(color = condition, fill = condition))+
  stat_cor(method = "pearson", label.x=6)+
    labs(title = "Correlation between VAS and MI Total score during continuous motion session", y = "MI
  scale_color_manual(values = c("Auditory" = "grey", "Heat" = "red"))+
  theme_minimal()+
  scale_fill_manual(values = c("Auditory" = "grey", "Heat" = "red"))

gg_cor_ContinuKMI <- ggplot(cor_var_ContinuKMI, aes(x=VAS, y=ContinuMI_KMI, color = condition, shape =
  geom_point() +
  geom_smooth(method=lm, aes(color = condition, fill = condition))+
  stat_cor(method = "pearson", label.x=6)+
    labs(title = "Correlation between VAS and KMI score during continuous motion session", y = "KMI Sco
  scale_color_manual(values = c("Auditory" = "grey", "Heat" = "red"))+
  theme_minimal()+
  scale_fill_manual(values = c("Auditory" = "grey", "Heat" = "red"))

gg_cor_ContinuVMI <- ggplot(cor_var_ContinuVMI, aes(x=VAS, y=ContinuMI_VMI, color = condition, shape =
  geom_point() +
  geom_smooth(method=lm,  aes(color = condition, fill = condition))+
  stat_cor(method = "pearson", label.x=6)+
    labs(title = "Correlation between VAS and VMI score during continuous motion session", y = "VMI Sco
  scale_color_manual(values = c("Auditory" = "grey", "Heat" = "red"))+
  theme_minimal()+
  scale_fill_manual(values = c("Auditory" = "grey", "Heat" = "red"))

print(gg_cor_ContinuTotal)
```

Visualize the data

```
## `geom_smooth()` using formula = 'y ~ x'
```

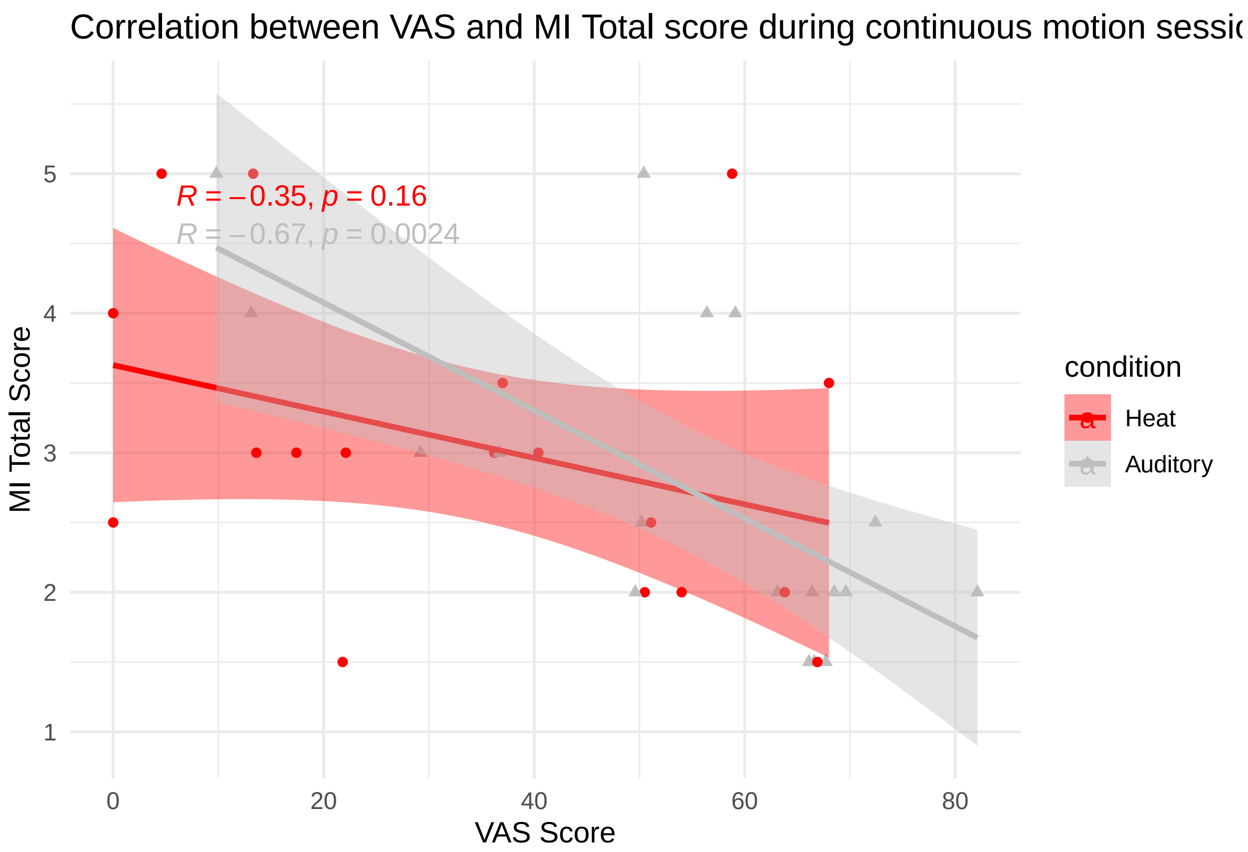


```r
print(gg_cor_ContinuKMI)
```

```
## 'geom_smooth()' using formula = 'y ~ x'
```

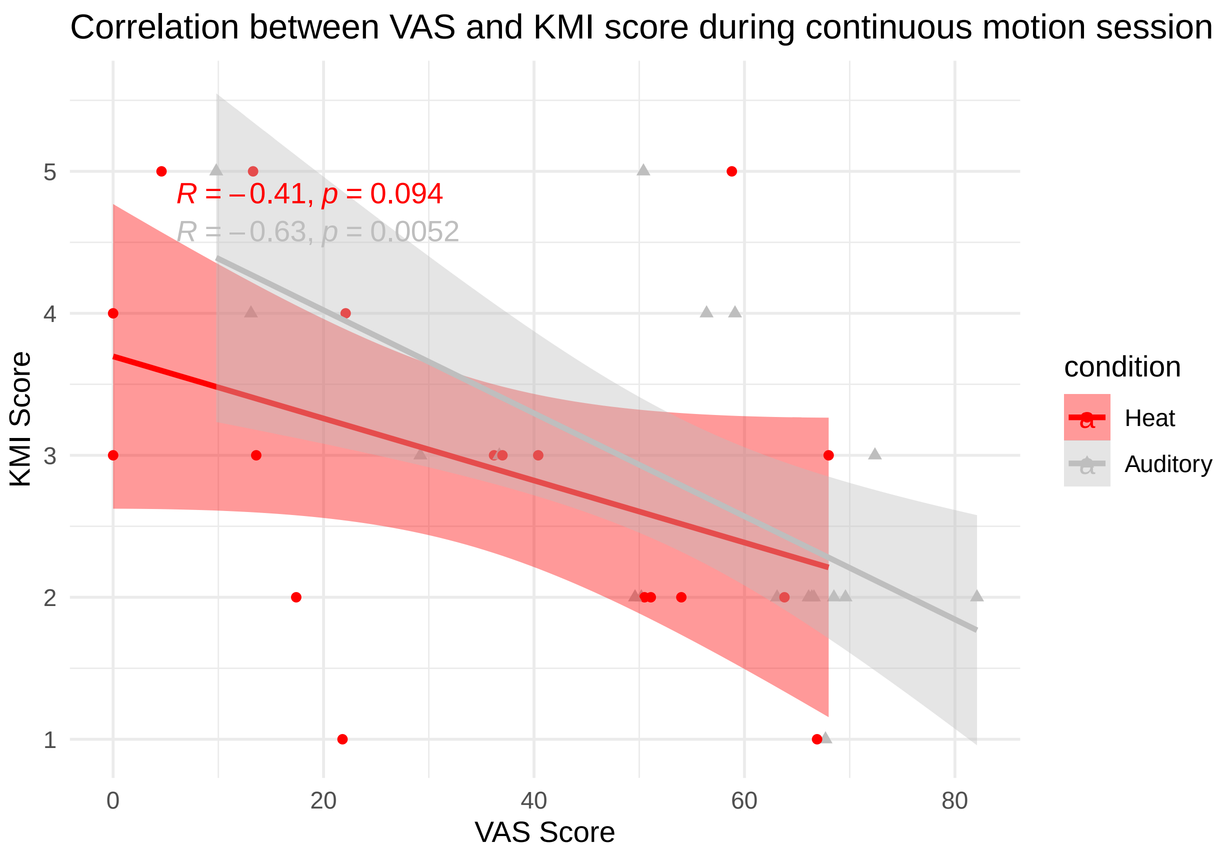


```
print(gg_cor_ContinuVMI)
```

```
## `geom_smooth()` using formula = 'y ~ x'
```

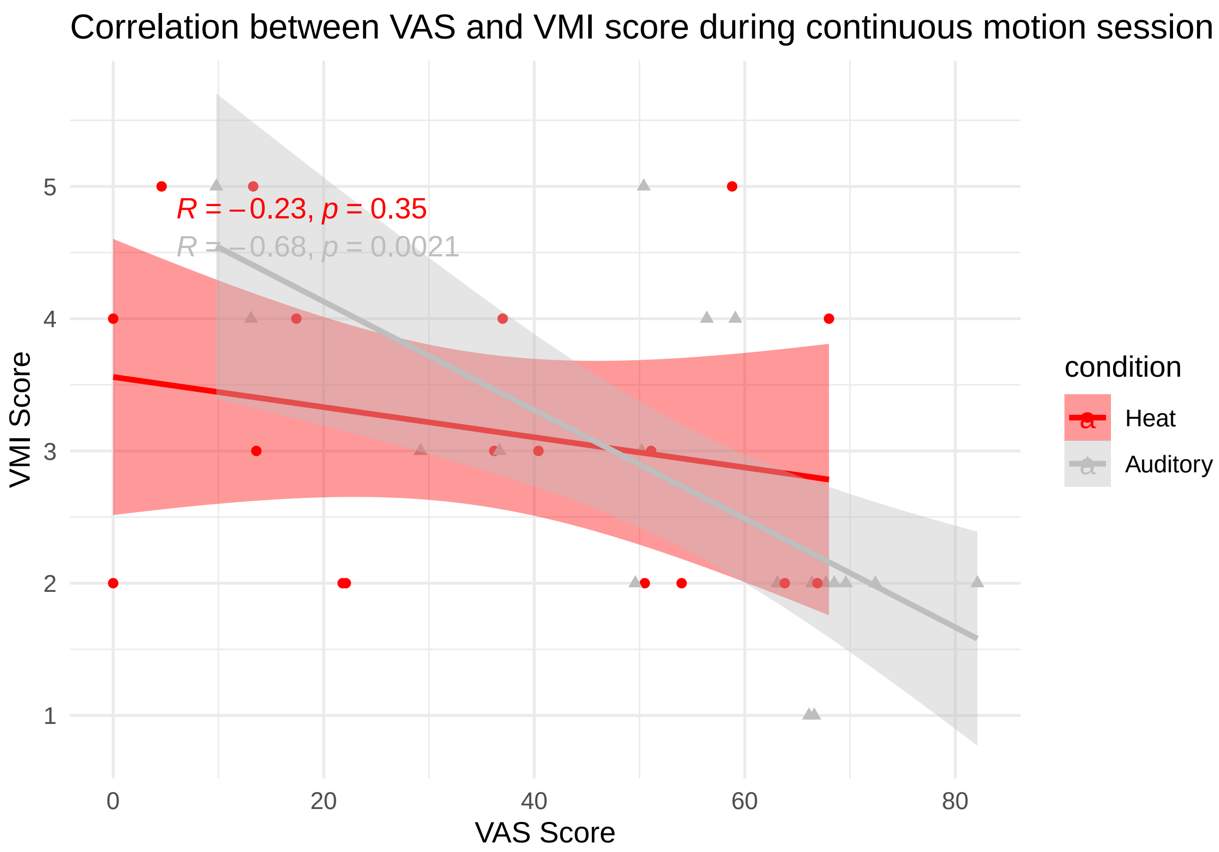

Conclusion

The results of the analysis indicate that there is a significant negative correlation between the VAS score and the MI abilities during continuous motion session, only for the auditory condition. The auditory unpleasant stimulus has a high negative impact on MI abilities compared to the heat and Control conditions.

Confounding factors analysis

Add KVIQ total score

```r
data$KVIQ_Total <- (data$KVIQ_V + data$KVIQ_K)
```

Create a new dataframe with factors

```r
# Create a dataframe for the Motor Imagery Total Score
Confund <- data %>%
  select(ID, Age, Sex, BMI, KVIQ_K, KVIQ_V, KVIQ_Total, IPAQ_MET)
```

Merge Confund and MI

```r
# Merge Confund dataframe with MI_Total
Confund_MI_Total <- merge(Confund, MI_Total, by = "ID")

Confund_MI_Total$ID <- as.numeric(Confund_MI_Total$ID)
Confund_MI_Total$BMI <- as.numeric(Confund_MI_Total$BMI)
Confund_MI_Total$KVIQ_K <- as.numeric(Confund_MI_Total$KVIQ_K)
Confund_MI_Total$KVIQ_V <- as.numeric(Confund_MI_Total$KVIQ_V)
Confund_MI_Total$KVIQ_Total <- as.numeric(Confund_MI_Total$KVIQ_Total)
Confund_MI_Total$IPAQ_MET <- as.numeric(Confund_MI_Total$IPAQ_MET)
Confund_MI_Total$MI_TotalScore <- as.numeric(Confund_MI_Total$MI_TotalScore)
```

```r
# Merge Confund dataframe with ContinuMI_Total
Confund_ContinuMI_Total <- merge(Confund, ContinuMI_Total, by = "ID")

Confund_ContinuMI_Total$ID <- as.numeric(Confund_ContinuMI_Total$ID)
Confund_ContinuMI_Total$BMI <- as.numeric(Confund_ContinuMI_Total$BMI)
Confund_ContinuMI_Total$KVIQ_K <- as.numeric(Confund_ContinuMI_Total$KVIQ_K)
Confund_ContinuMI_Total$KVIQ_V <- as.numeric(Confund_ContinuMI_Total$KVIQ_V)
Confund_ContinuMI_Total$KVIQ_Total <- as.numeric(Confund_ContinuMI_Total$KVIQ_Total)
Confund_ContinuMI_Total$IPAQ_MET <- as.numeric(Confund_ContinuMI_Total$IPAQ_MET)
Confund_ContinuMI_Total$MI_TotalScore <- as.numeric(Confund_ContinuMI_Total$ContinuMI_TotalScore)
```

Analyse the confounding factors for discrete session

```r
library(lmerTest)

model <- lmer(MI_TotalScore ~ condition + Age + Sex + BMI + KVIQ_Total + IPAQ_MET + (1 | ID), data = Co

summary(model)
```

```
## Linear mixed model fit by REML. t-tests use Satterthwaite's method [
## lmerModLmerTest]
## Formula: MI_TotalScore ~ condition + Age + Sex + BMI + KVIQ_Total + IPAQ_MET +
##     (1 | ID)
##    Data: Confund_MI_Total
##
## REML criterion at convergence: 143.1
##
## Scaled residuals:
##         Min          1Q      Median          3Q         Max
## -1.81420654 -0.51525303  0.01593254  0.51486884  1.63334836
##
## Random effects:
##  Groups   Name        Variance  Std.Dev.
##  ID       (Intercept) 0.6731339 0.8204474
##  Residual             0.2415577 0.4914852
## Number of obs: 54, groups:  ID, 18
##
```

```
## Fixed effects:
##                       Estimate   Std. Error           df  t value   Pr(>|t|)
## (Intercept)        2.728126e+00 3.234279e+00 1.202055e+01  0.84350 0.41541719
## conditionHeat     -2.500000e-01 1.638284e-01 3.400000e+01 -1.52599 0.13626278
## conditionAuditory -6.944444e-01 1.638284e-01 3.400000e+01 -4.23885 0.00016246
## Age                7.837279e-03 4.507782e-02 1.200000e+01  0.17386 0.86487313
## SexH               6.812295e-01 5.556604e-01 1.200000e+01  1.22598 0.24372090
## BMI               -7.276313e-02 1.093553e-01 1.200000e+01 -0.66538 0.51839083
## KVIQ_Total         4.189305e-02 5.551331e-02 1.200000e+01  0.75465 0.46501277
## IPAQ_MET           2.645994e-05 4.665273e-05 1.200000e+01  0.56717 0.58105497
##
## (Intercept)
## conditionHeat
## conditionAuditory ***
## Age
## SexH
## BMI
## KVIQ_Total
## IPAQ_MET
## ---
## Signif. codes:  0 '***' 0.001 '**' 0.01 '*' 0.05 '.' 0.1 ' ' 1
##
## Correlation of Fixed Effects:
##             (Intr) cndtnH cndtnA Age    SexH   BMI    KVIQ_T
## conditionHt -0.025
## condtnAdtry -0.025  0.500
## Age         -0.339  0.000  0.000
## SexH         0.286  0.000  0.000  0.239
## BMI         -0.688  0.000  0.000 -0.018 -0.614
## KVIQ_Total  -0.637  0.000  0.000 -0.050 -0.045  0.051
## IPAQ_MET     0.205  0.000  0.000  0.146  0.244 -0.373 -0.141
## fit warnings:
## Some predictor variables are on very different scales: consider rescaling
```

Analyse the confounding factors for continuous session

```r
model2 <- lmer(ContinuMI_TotalScore ~ condition + Age + Sex + BMI + KVIQ_Total + IPAQ_MET + (1 | ID), d[illegible]

summary(model2)
```

```
## Linear mixed model fit by REML. t-tests use Satterthwaite's method [
## lmerModLmerTest]
## Formula: ContinuMI_TotalScore ~ condition + Age + Sex + BMI + KVIQ_Total +
##     IPAQ_MET + (1 | ID)
##    Data: Confund_ContinuMI_Total
##
## REML criterion at convergence: 163.7
##
## Scaled residuals:
##         Min          1Q      Median          3Q         Max
## -2.40824457 -0.51069145  0.06084209  0.42661310  2.51330113
##
```

```
## Random effects:
##  Groups   Name        Variance  Std.Dev.
##  ID       (Intercept) 0.7325764 0.8559068
##  Residual             0.4212963 0.6490734
## Number of obs: 54, groups:  ID, 18
##
## Fixed effects:
##                         Estimate    Std. Error            df   t value Pr(>|t|)
## (Intercept)        2.176428e+00  3.481727e+00  1.203094e+01  0.62510 0.543580
## conditionHeat     -1.111111e-01  2.163578e-01  3.400000e+01 -0.51355 0.610886
## conditionAuditory -4.166667e-01  2.163578e-01  3.400000e+01 -1.92582 0.062522 .
## Age                8.717639e-02  4.851616e-02  1.200000e+01  1.79685 0.097554 .
## SexH               1.212415e+00  5.980437e-01  1.200000e+01  2.02730 0.065439 .
## BMI               -6.604204e-02  1.176965e-01  1.200000e+01 -0.56112 0.585041
## KVIQ_Total        -1.704233e-02  5.974762e-02  1.200000e+01 -0.28524 0.780325
## IPAQ_MET           3.104364e-05  5.021119e-05  1.200000e+01  0.61826 0.547959
## ---
## Signif. codes:  0 '***' 0.001 '**' 0.01 '*' 0.05 '.' 0.1 ' ' 1
##
## Correlation of Fixed Effects:
##             (Intr) cndtnH cndtnA Age    SexH   BMI    KVIQ_T
## conditionHt -0.031
## condtnAdtry -0.031  0.500
## Age         -0.339  0.000  0.000
## SexH         0.286  0.000  0.000  0.239
## BMI         -0.688  0.000  0.000 -0.018 -0.614
## KVIQ_Total  -0.636  0.000  0.000 -0.050 -0.045  0.051
## IPAQ_MET     0.205  0.000  0.000  0.146  0.244 -0.373 -0.141
## fit warnings:
## Some predictor variables are on very different scales: consider rescaling
```

Conclusion

There was no significant effect of the confounding factors on the MI Total Score during discrete and continuous session. The results indicate that the condition has a significant effect on the MI Total Score, but the confounding factors do not significantly influence the results.

Merge VAS scores

```
Merge_VAS <- merge(VAS_Exe,VAS_MI, by = c("ID", "condition"))
Merge_ContinuVAS <- merge(ContinuVAS_Exe,ContinuVAS_MI, by = c("ID", "condition"))
```

Plot the VAS scores

```
# Create a new dataframe for the VAS scores
VAS_scores <- Merge_VAS %>%
  select(ID, condition, VAS_Exe, VAS_MI) %>%
  pivot_longer(
```

```r
    cols = c(VAS_Exe, VAS_MI),
    names_to = "Tasks",
    values_to = "VAS"
  )
# Create a new dataframe for the continuous VAS scores
ContinuVAS_scores <- Merge_ContinuVAS %>%
  select(ID, condition, VAS_Exe, VAS_MI) %>%
  pivot_longer(
    cols = c(VAS_Exe, VAS_MI),
    names_to = "Tasks",
    values_to = "VAS"
  )
```

```r
labels <- c("Motor Execution", "Motor Imagery")

# Plot the VAS scores
Plot1 <- ggplot(VAS_scores, aes(x = Tasks, y = VAS, fill = condition)) +
  geom_boxplot(alpha = 0.4, outlier.shape = NA) +
  theme_minimal() +
  theme(plot.title = element_text(hjust = 0.5))+
  scale_fill_manual(values = c("Control" = "green", "Heat" = "red", "Auditory" = "grey"))+
  scale_color_manual(values = c("Control" = "green", "Heat" = "red", "Auditory" = "grey"))+
  scale_x_discrete(label = labels)

# Plot the continuous VAS scores
Plot2 <- ggplot(ContinuVAS_scores, aes(x = Tasks, y = VAS, fill = condition)) +
  geom_boxplot(alpha = 0.4, outlier.shape = NA) +
  theme_minimal() +
  theme(plot.title = element_text(hjust = 0.5))+
  scale_fill_manual(values = c("Control" = "green", "Heat" = "red", "Auditory" = "grey"))+
  scale_color_manual(values = c("Control" = "green", "Heat" = "red", "Auditory" = "grey"))+
  scale_x_discrete(label = labels)
```

Combine the plots

```r
ggarrange(Plot1, Plot2,
          labels = c("A", "B"),
          ncol = 1,  # Change to 1 column
          nrow = 2,  # Change to 2 rows
          common.legend = TRUE,
          legend = "bottom")
```

```
## Warning: Removed 8 rows containing non-finite outside the scale range
## (`stat_boxplot()`).

## Warning: No shared levels found between `names(values)` of the manual scale and the
## data's colour values.

## Warning: Removed 8 rows containing non-finite outside the scale range
## (`stat_boxplot()`).
```

```
## Warning: No shared levels found between `names(values)` of the manual scale and the
## data's colour values.

## Warning: Removed 7 rows containing non-finite outside the scale range
## (`stat_boxplot()`).

## Warning: No shared levels found between `names(values)` of the manual scale and the
## data's colour values.
```

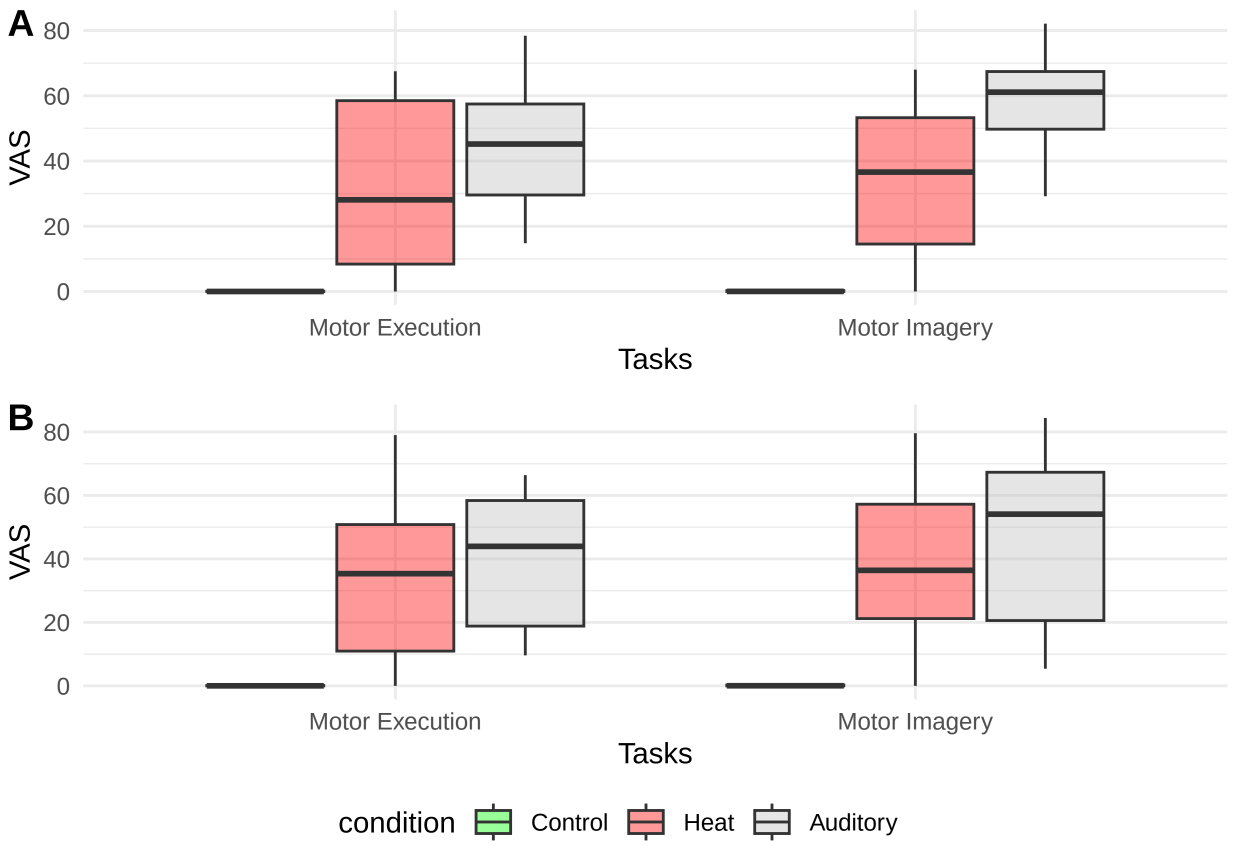

Supplement: S1 File — (PDF) [file pone.0321343.s003.pdf]
