# Supplementary material for: The impact of thermal and auditory unpleasant stimulus on explicit motor imagery in healthy individuals: An experimental study
Source: PLoS One. 2025 Sep 22;20(9):e0321343. doi: 10.1371/journal.pone.0321343 (PMC12453216; doi:10.1371/journal.pone.0321343)
Supplement: Attachment — Submitted filename: Response to Reviewers.docx [file pone.0321343.s004.docx]

Response to Reviewers

Dear ACADEMIC EDITOR, Thomas Rulleau,

Please take note of the changes made to the manuscript, highlighted in blue, in response to your suggestions. Our answers to your questions have been written in blue in the manuscript and below:

1. Please indicate the checklist on which your paper is based (CONSORT? STROBE? SPIRIT?).

After reviewing the EQUATOR website, we found the CRED-nf checklist to be the most appropriate for our experimental research design.

2. Change the title to explicitly mention the method.

As per your suggestion, we have revised the title to include "a controlled experimental study".

We thank you for your consideration.

Sincerely,
