# Supplementary material for: The impact of thermal and auditory unpleasant stimulus on explicit motor imagery in healthy individuals: An experimental study
Source: PLoS One. 2025 Sep 22;20(9):e0321343. doi: 10.1371/journal.pone.0321343 (PMC12453216; doi:10.1371/journal.pone.0321343)
Supplement: Attachment — Submitted filename: response to reviewers letter.docx [file pone.0321343.s005.docx]

RESPONSE TO REVIEWERS

We sincerely thank the editors and the two reviewers for their insightful and constructive suggestions, which have significantly contributed to improving the quality of our manuscript.

Please note that we have provided both a Clean Copy and a revised manuscript, with all comments and changes highlighted in blue. Our responses to the reviewers are also included in this letter and are marked in blue for clarity.

Reviewers' comments:

Reviewer's Responses to Questions

**Comments to the Author**

1. If the authors have adequately addressed your comments raised in a previous round of review and you feel that this manuscript is now acceptable for publication, you may indicate that here to bypass the “Comments to the Author” section, enter your conflict of interest statement in the “Confidential to Editor” section, and submit your "Accept" recommendation.

Reviewer #1: (No Response)

Reviewer #2: (No Response)

2. Is the manuscript technically sound, and do the data support the conclusions?

The manuscript must describe a technically sound piece of scientific research with data that supports the conclusions. Experiments must have been conducted rigorously, with appropriate controls, replication, and sample sizes. The conclusions must be drawn appropriately based on the data presented.

Reviewer #1: (No Response)

Reviewer #2: Yes

3. Has the statistical analysis been performed appropriately and rigorously?

Reviewer #1: (No Response)

Reviewer #2: Yes

4. Have the authors made all data underlying the findings in their manuscript fully available?

The [PLOS Data policy](http://www.plosone.org/static/policies.action#sharing) requires authors to make all data underlying the findings described in their manuscript fully available without restriction, with rare exception (please refer to the Data Availability Statement in the manuscript PDF file). The data should be provided as part of the manuscript or its supporting information, or deposited to a public repository. For example, in addition to summary statistics, the data points behind means, medians and variance measures should be available. If there are restrictions on publicly sharing data—e.g. participant privacy or use of data from a third party—those must be specified.

Reviewer #1: (No Response)

Reviewer #2: Yes

5. Is the manuscript presented in an intelligible fashion and written in standard English?

PLOS ONE does not copyedit accepted manuscripts, so the language in submitted articles must be clear, correct, and unambiguous. Any typographical or grammatical errors should be corrected at revision, so please note any specific errors here.

Reviewer #1: (No Response)

Reviewer #2: Yes

6. Review Comments to the Author

Please use the space provided to explain your answers to the questions above. You may also include additional comments for the author, including concerns about dual publication, research ethics, or publication ethics. (Please upload your review as an attachment if it exceeds 20,000 characters)

Reviewer #1: Reviewing PONE-D-25-10295_R1-1

I would thank the Editor, Doctor Thomas Rulleau for his invitation to perform this review of the manuscript entitled “The impact of thermal and auditory unpleasant stimulus on motor imagery in healthy individuals: an experimental study » by Cohen-Aknine Gabriel, Pionnier Raphaël, Pr Mottet Denis and Pr Dupeyron Arnaud.

I would like to thank the Authors for submitting their article. I acknowledge that a previous revision was performed. Based on the revised version of the manuscript, I formulated comments structured in 3 parts : majors, additionals and comments on the text that could further improve the quality of the manuscript.

Major comments:
- Regarding the conditions of the exposure. Is there any chance that pleasant stimuli would have resulted in MI improvement ? This is not discussed whereas it could have been. The rational of using the 3 groups (and not more) could be elaborated in the manuscript.

We would like to thank Reviewer 1, Dr Sébastien Mateo, for his suggestion. Our selection of three groups was based on our objective of focusing on unpleasant sensory stimuli and their impact on motor imagery vividness, particularly in relation to clinical pain and rehabilitation applications. Other researchers have indeed described the effects of pleasant, neutral and unpleasant stimuli on motor imagery vividness. For example, Bywaters et al. (10.1080/09658210444000160) have demonstrated that affective states involving pleasant and unpleasant imagery modify motor imagery vividness. These findings were further supported by a study by Wriessnegger et al. (10.1016/j.bandc.2018.07.006), which used functional near-infrared spectroscopy to detect increased cortical activity during negative, unpleasant imagery related to painful experiences. Consequently, we have added a rationale section to the 'Materials and Methods' explaining our choice of the three groups, as follows:

Line 201-206: ”Our three-group comparison, which focused solely on unpleasant stimuli and excluded a pleasant group, was justified for two main reasons. Firstly, this approach aligns with previous studies that examined the impact of affective states (both pleasant and unpleasant) on the vividness of explicit motor imagery (46,47). Secondly, our study focuses on negative affective states related to painful experiences, as defined by the IASP (1).”

- Result part should be revised with not corresponding text and Table. This discrepency result in a text hard to follow and understand.

Thank you for your feedback. Following your suggestion, we have revised the Results section and added a new table, Table S1, in Appendix 1. We apologize for the error regarding the questionnaire name, which was mistakenly referenced from another study. The necessary corrections have been made in line with the reviewer's suggestions.

The results are now presented in two distinct parts: one for the discrete sessions and one for the continuous sessions. These results account for the total scores, as well as the kinesthetic and visual modalities separately. For greater clarity, all the results are summarized in Table S1, which has been included in Appendix 1.

- Title could include an explicite mention of the pilot or proof-of-concept study rather or in extension to experimental study.
In line with this comment, please mention that no sample size was computed.

Thank you for these suggestions. Our decision not to calculate the sample size was based on previous experimental studies focused on pain models, such as those involving thermal painful stimulation. The following reviews reference these studies: Bank et al. (10.1002/j.1532-2149.2012.00186.x), Devecchi et al. (10.1097/J.PAIN.0000000000002819), and Izadi et al. (10.3389/fnhum.2022.863741). However, some authors have reported guidelines related to sample size calculation, such as Serdar et al. (10.11613/BM.2021.010502). For experimental studies, these authors recommend a sample size of 10 to 20 subjects for group comparisons. The rationale for the absence of a sample size calculation has been added to the "Materials and Methods" section as follows:

Line 109-121: “No sample size calculation was provided. However, some authors recommend a sample size of 10 to 20 subjects for similar studies (37).”

- Reading of the text and figures don’t explicitely inform about the movement speed between the continuous and discrete wrist flexion – extensions. Please clarify whether the movement speed was similar and controlled between conditions ;

We agree with this important point regarding the movement speed performed by the participants. Additional details have been added to the "Materials and Methods" section as follows:

Line 157-163: “Prior to the experiment, a training session was conducted to familiarize participants with the movement, consisting in performing a full range of motion wrist back-and-forth movement at a regular and consistent speed for four seconds, without acceleration or deceleration. Based on the kinesiology of the movement (45), the full range of motion of the wrist is 120°, with back-and-forth joint movement; for a total of 240° in four seconds. Therefore, we can estimate that the movement has an arbitrary expected speed of 60° per second.

Additional comments:
- In the title, abstract and the rest of the manuscript, it could be explicitely state that the authors refers to explicit motor imagery. Also, please use MI vividness instead of ability.

Thank for this indication. Additional precisions about the type of motor imagery and the use of “vividness” were used to replace the term “modality”, in accordance with the suggestion and the following article : https://doi.org/10.1016/j.neures.2018.12.005

In addition, the title was changed as suggested:

Line 1-3:” The impact of thermal and auditory unpleasant stimulus on explicit motor imagery in healthy individuals: an experimental study”

- Please elaborate the outcome of the measure since impairing motor imagery is not engouht precise.

We only used the two KVIQ 5-point Likert subscales for kinesthetic and visual motor imagery to measure motor imagery vividness. We did not use other tools. All detailed results are provided in Appendix 1 Table S1.

- It seems to be essential to dinstiguish different aspects separating those refering to the delay in experiencing disease such as pain (and inducing potentially long term plasticity changes) to those elicited by MI training during a single session (i.e., more likely associated to short term changes).

Thank you for this significant suggestion. Additional information has been added to the "Clinical Implications" section to clarify the distinction between the short- and long-term effects of motor imagery training. According to Suso-Martí et al. and Cuenca-Martinez et al., motor imagery training may require repetition and a prolonged duration to decrease pain intensity. The following sentence was added in the Discussion section:

Line 391-393:” However, these proposals should be approached with caution given the one-day experiment of the motor imagery tasks in the protocol of our study.

Additionally, the sentence below was added to the Clinical Implications section:

Line 469-471:” Previous authors have suggested that significant dosage or duration of motor imagery training is required to effectively decrease pain intensity (77,78), suggesting caution in interpreting the results.”

- Some details could be added regarding the instruction provided to the participant during MI to complete the control of potential experimental bias.

We have rewritten the instructions and description of the procedure for greater readability.

Line 157-165:” Prior to the experiment, a training session was conducted to familiarize participants with the movement, consisting in performing a full range of motion wrist back-and-forth movement at a regular and consistent speed for four seconds, without acceleration or deceleration. Based on the kinesiology of the movement (45), the full range of motion of the wrist is 120°, with back-and-forth joint movement; for a total of 240° in four seconds. Therefore, we can estimate that the movement has an arbitrary expected speed of 60° per second.

The instructions for the discrete and continuous sessions are described in parts 1 and 2 below.

And line 170-189:

“Part 1: discrete session

With their wrists fully flexed, as described above, the participants were instructed to perform a single, full-range-of-motion wrist movement in the sagittal plane (from a fully flexed position to a fully extended position) and then return to the starting position within a 4-second time window, consistent with the training session. This procedure is known as the discrete session. The session began with motor execution, followed by a motor imagery task. During the motor imagery task, participants had to imagine performing the same movement. This procedure was repeated 20 times for each task (Fig. 2).

Part 2: continuous session

Participants were instructed to perform repeated, 4-second, full-range-of-motion, back-and-forth wrist movements in the sagittal plane within a 25-second time window, consistent with the training session. They started in the same position as in part 1. This procedure is known as the continuous session. Participants were instructed to repeat the four-second movement cycle until the word "STOP" appeared on the screen. The session began with motor execution, followed by a motor imagery task. During the motor imagery task, participants had to imagine performing the same movements within the same time frame. This procedure was repeated three times for each task (Fig. 2).”

- Did the authors face adverse effect with the use of painful thermal condition ? Please reporte this in the manuscript (in the result part) as long as the procedure to manage such event (in the method).

Thank you for this important suggestion. Of course, all participants were closely supervised during each session to prevent any adverse effects. If any adverse events were detected, the procedure was immediately stopped. To date, no participants have reported any adverse events.

In accordance with Reviewer 1’s suggestion, we have added the following sentences to the "Material and Methods" section:

Line 116-118:” Participants were excluded from the study if they were unable to perform the procedure described below and/or because of a dysfunction of any equipment used in the protocol or in case of adverse events.

and line 213-217:” The initial duration of the hot water immersion was based on a study by Granot et al. (48), in which participants experienced a 60-second immersion. However, due to the length of our protocol and to minimize the risk of adverse events, we reduced the immersion time to 30 seconds before the tasks began.”

Line 224-225:” Finally, the participants were asked to report any adverse events related to the study protocol, whether immediate or delayed.”

Additionally, the absence of adverse events was reported in the "Results" section:

Line 347-348:” Throughout the study, none of the participants reported any adverse events, whether during the discrete or continuous session.”

- In the outcomes, authors state using of the Likert scale of the KVIQ and report result of the MIQ which is confusing and should be corrected.
Pleasr also report the unpleasant mesure of the condition in the method to describe the procedure and report results in the corresponding part.

We sincerely apologize for this oversight. The MIQ was indeed used in another study, and reusing the R codes caused this error. We have made the necessary corrections.

Comment on the text.
In the intro, ref 11, 12 about the rational of using MI without pain. Moseley et al., have shown that in case of complex regional pain syndrome, a non-negligible (~50%) of participants did showed an increase in pain intensity. Please elaborate.

Thank you for the opportunity to discuss the effectiveness of motor imagery and the need for caution regarding its immediate effects on specific populations. In response to the reviewer's comment, we revised the "Introduction" section to include findings from Moseley et al. 2008 (10.1002/art.23580), noting that about 50% of participants with complex regional pain syndrome experienced increased pain intensity. The following elaboration has been added to the "Introduction" section:

Line 65-68:” However, Moseley et al. (13) showed an increase in pain intensity during motor imagery training for patients with chronic pain complains, such as complex regional pain syndrome. This finding may mitigate the promising results of this therapy.”

Line 70: did the authors referes to implicit or explicit MI ? Please elaborate.

This explanation refers to both implicit and explicit MI. We have made the corresponding changes:

Line 75-77:” However, some studies suggest that the experience of pain may reduce implicit (24) and explicit MI vividness (25) in individuals with chronic pain, although these findings vary depending on the type of pain (26).”

Line 77-79 Results of Ref 31 could be presented here.

Precision about the results of these experiments was added in the "Introduction" section as follows:

Line 82-88:" A previous study showed that experimental pain could interfere with MI processes in healthy individuals (32In this study, the authors used a phasic pain stimulus involving electrical stimulation of the fifth digit and the knee during a thumb flexion/extension motor imagery task. The results showed a reduction in corticospinal excitability during motor imagery tasks regardless of location of the pain stimulus. However, to our knowledge, few information is available about whether the type of pain and its different dimensions (affective, cognitive and sensory) influence MI vividness.”

Line 81-88 : The rational of comparing discrete vs continuous MI could be elaborate. The authors could find some information in the recent paper by : Suica, Z., Behrendt, F., Gäumann, S., Gerth, U., Schmidt-Trucksäss, A., Ettlin, T., & Schuster-Amft, C. (2022). Imagery ability assessments : A cross-disciplinary systematic review and quality evaluation of psychometric properties. BMC Medicine, 20(1), 166. https://doi.org/10.1186/s12916-022-02295-3 Also, please revise the term modality which refers to visual or kinaesthetic and seems not appropriate here. In this part, the clinical implication of this work could be added.
Participant, please elaborate the choice of the KVIQ as MI questionnaire vs other questionnaires validated in French (i.e., MIQ-3 2020, 10.1051/sm/2019035).

Thank you for bringing this interesting article to our attention. We only discovered it after completing our study. Indeed, the term "modality" can be confusing in the context of discrete and continuous motor imagery. We developed our rationale based on the article by Rimbert et al., which, to our knowledge, is the only study that has evaluated these two types of sessions. To maintain consistency with the previous study, we renamed the term "modality" to "session" retained the terms "continuous" and "discrete" motor imagery.

Although the MIQ-RS (Movement Imagery Questionnaire-Revised Short Version) appears to be a more reliable tool for assessing motor imagery capabilities, the French versions of the MIQ-RS and the KVIQ (Kinesthetic and Visual Imagery Questionnaire) have similar psychometric properties (Malouin et al. showed that the KVIQ ICC ranges from 0.72 to 0.81, with internal consistencies of 0.89 and 0.87 for the visual and kinesthetic modalities, respectively. In contrast, Loison et al. showed that the MIQ-RS ICC ranges from 0.68 to 0.78, with an internal consistency of 0.90. Therefore, while Suica et al. recommend the MIQ-RS, they note that the KVIQ remains the most widely used questionnaire in medical settings. Consequently, we consider the KVIQ the gold standard in clinical assessment.

Procedure : please revise the word modality which is inappropriately used.

We changed the term “modality” with “vividness” to describe motor imagery abilities as suggested.

A Figure could be helpful to better understand the experimental procedure.

To bring more clarity, we have changed the Protocol Design figure (Fig 2).

Line 150-1 : there was no specific instructions regarding visual or kinaesthetic motor imagery modalities for [MI]. The term modality is used appropriately here. The question is rather about the control of the MI : how was it perform in this condition ?

Thank you for raising this important question. Initially, we planned to use EEG in this study. However, due to data export issues during offline processing, we were unable to use the EEG data. Participants were instructed only to perform motor imagery in their chosen modality, either visual or kinesthetic. We trusted the participants, who were university students, to follow these instructions accurately.

Part 1 – 2. In addition to a new figure, this part is quite hard to follow with motor execution and imagery introduced before part 1. Please revise this section. It appears that there is figures for instruction design and protocol. These figures appears after the text and may be introduced earlier.

Thank you for the suggestion to improve readability. We have moved Figures 1, 2 and 3 to an earlier section of the document and changed the associated explanations.

Additionally, we have included more information in the Materials and Methods section.

Line 142-156:” Procedure

Participants were seated in a chair in front of a computer in a relaxed position with their forearms resting on the armrests and their elbows naturally flexed in pronation, so that the wrists were fully flexed and relaxed. The procedure was divided in into two sessions: a discrete session consisting of single executed followed by imagined wrist movements, and a continuous session consisting of repeated executed followed by imagined wrist movements. All participants began with the discrete session, followed by the continuous session. Since all participants were right-handed (Table 1), the movement was executed and imagined using their dominant right hand against gravity due to the action of the extensor muscles. The instructions were displayed on a screen using PsychoPy software (version 2023.1.2) (43,44).

In both sessions, all participants performed "motor execution" followed by "motor imagery" tasks. During the motor execution and imagery tasks, a blank screen was displayed between the on-screen ‘GO’ and ‘STOP’ instructions. Before each task, general instructions were displayed to remind participants of the requirements (Fig. 1).”

To enhance clarity and facilitate a better understanding of the study protocol, we have revised and updated the figure, which is available in the "Materials and Methods" section (Figure 2).

Part 1. Regarding the position, it appears that the wrist flexion extension is performed against gravity (i.e., similarly to a MRC manual muscle testing score = 3/5). Is this correct ? If so, please add this information in the text.

Indeed, the movement was performed against gravity. This important protocol detail has been added to the "Materials and Methods / Procedure" section as follows:

Line 149-151:” Since all participants were right-handed (Table 1), the movement was executed and imagined using their dominant right hand against gravity due to the action of the extensor muscles.”

Part 2. Did the participant received an instruction for movement speed ? Please add this information in the text.

We thank the reviewer for this important suggestion regarding movement speed. As mentioned below, we have included this information in the "Material and Methods" section.

Line 157-165:” Prior to the experiment, a training session was conducted to familiarize participants with the movement, consisting in performing a full range of motion wrist back-and-forth movement at a regular and consistent speed for four seconds, without acceleration or deceleration. Based on the kinesiology of the movement (45), the full range of motion of the wrist is 120°, with back-and-forth joint movement; for a total of 240° in four seconds. Therefore, we can estimate that the movement has an arbitrary expected speed of 60° per second.

The instructions for the discrete and continuous sessions are described in parts 1 and 2 below.”

Conditions : please elaborate the rational for choosing each of them. Hot exposition seems to be presented lines 197 – 200. Is there recommended duration needed for exposure to hot water for the occurrence of pain ? Please elaborate.

Indeed, we aim to clarify the duration of immersion in hot water. Our protocol was based on the study by Valentini et al. (10.1016/j.neuroimage.2022.119143), where the authors did not specify the water immersion duration. However, their protocol was based on the study by Granot et al. (10.1016/j.pain.2007.06.029), in which the authors described a hot water immersion duration of 60 seconds. Due to the lengthy duration of our protocol, we decided to reduce this time to a 30-second immersion duration. We have added this clarification to the "Materials and Methods" section as follows:

Line 213-217:” The initial duration of the hot water immersion was based on a study by Granot et al. (48), in which participants experienced a 60-second immersion. However, due to the length of our protocol and to minimize the risk of adverse events, we reduced the immersion time to 30 seconds before the tasks began.

It could be more appropriate to start with detailing each condition and then specifying the order. Regarding the order, it is not clear how the dice was used to generate the random order in the conditions. Please elaborate.

As suggested by Reviewer 1, we changed the formulation concerning the randomization as follows:

Line 203-206:” The order of the conditions was randomized using a dice, leading to six task sequence designs.”

Additionally, we have included a figure (Fig. S1) in Appendix 1 to provide further clarity regarding the randomization process.

Line 174. The authors state “without unpleasant stimulus” which seems to refers to only auditory one but not to painful one. I suggest a revision of this sentence.

Thank you for these clarifications. We have revised the sentence as follows:

Line 205-206:” In the "control" condition, movement was executed and imagined in the absence of aversive auditory or painful thermal stimuli (see Appendix 1 Fig. S1).”

Primary outcome :
Is it only the effect of pain sensation which is examined ? I would suggest a revision as there are 3 different conditions. This could include the effect of conditions on MI vividness change (see my next comment).

As suggested, we have revised the Results section to present results into two parts following the order

Line 243-250:” The effect of conditions on explicit motor imagery vividness was assessed using KVIQ's 5-point Likert scale. The two kinesthetic (KMI) and visual (VMI) motor imagery subscales were recorded for each session (discrete and continuous), and at the end of each condition (control, heat and auditory). The total score was calculated as the mean of the two subscales.

Additionally, the level of unpleasantness was assessed after each condition and task using the same Visual Analogue Scale (VAS), which that was established at the beginning of the protocol to measure self-rated levels of stimulus discomfort.”

I also recommend a word changing MI ability into MI vividness using the Likert score of the KVIQ.

We have implemented this change throughout the entire manuscrit. Thank you for your suggestion, which aligns with the established literature nomenclature.

Statistics: Is there no additional exploratory analyse that can be performed relating the activity (IPAQ) and vividness score and separating the effect of gender ? Or regarding the MI vividness and an unpleasant measure of score (if any was measured) ? This latter would have been of particular interest for the present study. In addition, is there any differences regarding the MI ability. Are poor imagers more affected by conditions as compared to good imagers ?

After conducting the data analysis, we performed numerous statistical analyses. However, to avoid overwhelming the reader, we decided to remove some of these analyses from the article. Based on Reviewer 1's recommendation, we added more analyses to the supplementary materials in Appendix 2. These complementary analyses showed that there were no effects of the IPAQ, age, sex, BMI, or initial KVIQ score on motor imagery vividness, whether during discrete or continuous sessions.

Results :
245. I recommend moving at the end of the result the mention of the appendix 1.

This change has been made as recommended.

249. Is the significant difference reported only for the auditory condition vs the 2 others ? please elaborate. Table 3 (continuous condition) appears twice first in the discret condition and again in the continuous one. Understanding of the results is were hard with the presentation of the table. Please revise the result part for the main outcome.

The post hoc analysis revealed significant differences in the results for discrete session only between the auditory and normal conditions. There were no significant differences, however, between the auditory and heat conditions or between the heat and normal conditions. To simplify the interpretation of the findings, we chose not to specify these non-significant results.

However, to clarify the absence of difference, we have added this precision in the Result section:

Line 293-294:” However, there were no significant differences between the auditory and heat conditions or between the heat and control conditions.”

We sincerely apologize for the errors in the PDF document. In the Word version, Table 2 presents the results for the discrete session, while Table 3 presents the results for the continuous session. Unfortunately, these tables were inadvertently reversed in the PDF due to an oversight during formatting.

We have corrected this error to ensure accuracy. To improve clarity and readability, we have also merged the tables and included them in Appendix 1 (Table S1), supplemented with figures for better visualization of the results. Thank you for bringing this to our attention.

250. In the method, the authors refers to KVIQ and here to MIQ-RS. Please clarify what Likert scale was used to measure the MI vividness.
We apologize for this oversight. This error occurred because we reused old R code from another study that used the MIQ-RS. The necessary changes have been made.

258. There seems to be a typo “).” Before “Summaries”.

We have corrected this typo.

274. There seems to be an error in the reference.

Indeed, we apologize for this error due to figure referencing in the Word document. The necessary changes have been made.

Discussion.
See major comment.
Also, is there a chance that specifying instruction would have reduced the negative effect of the unpleasant stimulus ? Alternatively, the use of a pleasant or rhythmic auditory stimulus ?

We thank Reviewer 1 for this interesting suggestion. We explored the literature on the effects of verbal instruction on induced unpleasant or painful stimuli and motor imagery (MI) vividness. The study by den Hollander et al. (10.1111/pme.12836) found no effect of verbal instruction on the perception of pain. However, Atlas's review (10.1146/annurev-neuro-101822-122427) showed that verbal instruction can alter expectations, create anticipation, and influence aversive or painful responses. Unfortunately, we did not find any studies that specifically explore the effects of verbal instructions on MI vividness.

Hasegawa et al. studied the influence of different types of instructions on vividness and somatosensory activity. However, because they compared verbal, visual, and execution instructions, their results do not provide robust conclusions about the effect of verbal instruction alone on motor imagery vividness.

The study by Wriessnegger et al. (10.1080/08990220.2017.1368476) has shown that affective states, such as pleasant or unpleasant emotions, can change brain oxygenation during kinesthetic motor imagery. This study has been added to the "Materials and Methods" section as presented above.

The authors differentiate the cognitive and affective aspect of pain and explain their results (decrease of MI vividness after auditory condition). But the auditory condition could alter MI vividness because of an increase in the cognitive load while maintaining engaged in MI vividness. Please elaborate why the results are related to affective aspect more than cognitive on ?

Thank you for your feedback on this section of the discussion. It seems that this paragraph was not clear enough. We intended to suggest that the aversive auditory stimulus is more related to the cognitive-evaluative or motivational-emotional dimensions of pain than to the sensory-discriminative dimension, which is associated with precise localization.

We have rewritten the paragraph to provide a clearer explanation of our perspective. We drew on literature regarding both the cognitive-evaluative and motivational-emotional dimensions of pain, without favoring one over the other. We hope this revision clarifies our viewpoint:

Line 360-931:” In our study, we used two unpleasant stimuli to differentiate distinct aspects of pain, contrasting a stimulus without a localized component (aversive auditory stimulus) with a conventional experimental pain model (thermal heat stimulus), which provides a precise and unilateral localization related to the sensory-discriminative dimension of pain. The aversive auditory stimulus is likely more related to the cognitive-evaluative or motivational-emotional dimension due to its generally unpleasant characteristics and the global nature of the stimulus. In contrast, the thermal heat stimulus integrates the sensory-discriminative dimension, which is related to more localized unpleasant characteristics. On the one hand, with respect to the cognitive-evaluative dimension, previous research has shown that explicit motor imagery elicits high cognitive demand (12,54). In addition, studies have shown that motor imagery relies on working memory storage and induces mental fatigue (55) whether during prolonged implicit (56) or explicit motor imagery (57). Furthermore, research has shown a relationship between working memory and the perception of aversive sensations in response to unpleasant stimuli (58,59).

On the other hand, regarding the motivational-emotional dimension, however, some researchers have compared aversive auditory stimuli to a tinnitus-like sensation, which has been shown to activate cortical areas involved in attention and negative emotional responses (60). Studies have also shown that aversive stimuli, such as auditory stimulus, elicit amygdala responses (61) related to negative emotions, such as fear and anxiety (62). Furthermore, aversive auditory stimuli have been associated with conditioned threats and observed changes in alpha brain oscillations (63). Unpleasant and threatening situations are involved in motivational behaviors, as described in fear-avoidance models (64,65). Finally, a recent neurophysiological review proposed two brain pathways: one related to thalamo-somatosensory tracts involved in sensory-discriminative processing and another related to thalamo-prefrontal and cingulate tracts involved in cognitive-evaluative or motivational-emotional processing (66). Our results suggest that the cognitive-evaluative and motivational-emotional dimensions of pain may be more related to impair explicit motor imagery vividness, when assessed using an auditory stimulus. However, these proposals should be approached with caution given the one-day experiment of the motor imagery tasks in the protocol of our study.”

311. Please revise this statement since only a single session would have been performed.

We have taken these suggestions into consideration and added the following sentence:

Line 389-391:” However, these proposals should be approached with caution given the one-day experiment of the motor imagery tasks in the protocol of our study.”

364-6 this could be summarized in the result part.

We added the results of the motor imagery subdimensions, such as KMI and VMI, to the Results section. We also reorganized the section to be more consistent with the two parts of the study (discrete and continuous sessions).

Line 287-329:”Discrete motor imagery

KVIQ total score

There were significant statistical differences between conditions (p = 0.002). Post-hoc analysis revealed that the auditory stimulus decreased explicit motor imagery vividness compared to the control condition (p = 0.001) with a large effect size (d = 0.703) (Fig. 4). However, there were no significant differences between the auditory and heat conditions or between the heat and control conditions.

KVIQ Kinesthetic score

There were significant statistical differences between conditions (p = 0.005). Post-hoc analysis revealed that the auditory stimulus decreased explicit kinesthetic motor imagery vividness compared to the control condition (p = 0.037) with a large effect size (d = 0.605), and compared to the heat condition (p= 0.045) with a large effect size (d= 0.601) (Fig. 4).

KVIQ Visual score

There were significant statistical differences between conditions (p = 0.007). Post-hoc analysis revealed that the auditory stimulus decreased explicit visual motor imagery vividness compared to the control condition (p = 0.021) with a large effect size (d = 0.699) (Fig. 4).

Continuous motor imagery

Total KVIQ score

There were no significant statistical differences between conditions (p = 0.645), indicating that the unpleasant conditions did not impair explicit motor imagery vividness during the continuous motor imagery task.

KVIQ Kinesthetic score

There were no significant statistical differences between conditions (p = 0.663), indicating that the unpleasant conditions did not impair explicit kinesthetic motor imagery during the continuous motor imagery task.

KVIQ Visual score

There were no significant statistical differences between conditions (p = 0.103), indicating that the unpleasant conditions did not impair explicit visual motor imagery during the continuous motor imagery task.”

Please note that Figure 4 presents all the KVIQ scores and subscores.

366-9 this could be introduced earlier in the manuscript in the method and results. It could be added in the Table 1.

This point is indeed important, as the level of unpleasantness reflects the consistency of our experimental protocol. While the results are clear, we have added a concise paragraph to summarize the findings and included a figure for clarity (Fig. 5).

Line 331-337:” Levels of unpleasantness

Unpleasant VAS scores were significantly higher in aversive and heat conditions than in the control condition, in all sessions and for all tasks (p < 0.001) with large effect sizes (between 0.777 and 0.885). Post hoc analysis revealed that the auditory aversive stimulus led to higher VAS scores for discrete motor imagery than the heat pain stimulus (p = 0.008) with a large effect size (d = 0.672) (Fig. 5).”

Additionally, we have made the following additions to the Materials and Methods section:

Line 248-250: Additionally, the level of unpleasantness was assessed after each condition and task using the same Visual Analogue Scale (VAS) score that was established at the beginning of the protocol to measure self-rated levels of stimulus discomfort.”

385-7. I would rather temper this statement. On one hand, the result is obtained from a pilot study. On the other hand, the choice of the exercises (either continuous or intermittent) should be adapted to rehabilitation goal. It seems relevant to imagine continuous movement for walking but much less for balance exercises or grasping movement.

This point is indeed of particular importance, and our results should be considered with caution in the context of clinical pain and rehabilitation. We have made the appropriate changes as follows:

Line 457-463:” Therefore, personalized explicit motor imagery therapy is recommended according to rehabilitation goals and continuous sessions may be considered, based on our results. However, our results were assessed in an experimental design study, thus, longitudinal evaluation should be conducted to provide a more robust therapeutic approach. Additionally, our results suggest that the affective and cognitive dimensions of pain, as measured by an aversive auditory stimulus, impair the vividness of explicit motor imagery specifically in discrete sessions.”

Figures 4 and 5. I recommend to add the individual points of the 18 participants in the boxplots.

We thank Reviewer 1 for this suggestion. The participants' data points have been added to the boxplot as recommended.

Additional response.

Please note that the term "normal" condition has been replaced by "control."

I wisk good luck to the author for the revision of their manuscript.
Yours sincerely,
Dr Sébastien Mateo.

Reviewer #2: I thank the editor for inviting me to review this manuscript.

This manuscript presents a controlled experimental study investigating the impact of unpleasant stimuli on motor imagery abilities in a healthy population. The originality of this study lies in testing two different pain induction paradigms: an aversive auditory stimulus, which targets the affective component of pain and is fairly representative of the sensations experienced in chronic pain conditions, and a classical thermal stimulus, commonly used in the majority of laboratory pain induction protocols.

The study is clear and well-structured. The introduction provides an overview of recent literature in the field, the manuscript is easy to follow. The objective and hypotheses are defined and well-articulated. Although the work is fundamentally theoretical, its clinical implications are significant and thoroughly discussed. The limitations of this work are also clearly stated.

There are several minor comments to address, notably to the methods section.

Comment 1 Total score. I understand the methodological choice not to analyze the imagery modalities (visual and kinesthetic) separately, and to compute a total score, given the absence of specific instructions on how to perform the imagery. However, I question the relevance of adding the scores from the two subscales to obtain a total score out of 10, rather than calculating an average, which would have kept the results within the original 1-to-5 scale of the KVIQ. This would have made score interpretation easier by maintaining consistency with the original structure of the scale. Indeed, median scores around 4.5 remain difficult to interpret: it is not entirely clear whether the movement was "well" imagined, even though the reported imagery abilities suggest good quality imagery.

We thank Reviewer 2 for their suggestions. We have made the corresponding changes and calculated a total score based on the mean of the Visual and Kinesthetic subscores, which we believe makes the results more readable. We have added specification about the total score calculation in the Methods section as follows:

Line 243-247:” The effect of conditions on explicit motor imagery vividness was assessed using KVIQ's 5-point Likert scale. The two kinesthetic (KMI) and visual (VMI) motor imagery subscales were recorded for each session (discrete and continuous), and at the end of each condition (control, heat and auditory). The total score was calculated as the mean of the two subscales.”

Comment 2. Modality of motor imagery instructions. In the Methods section, it is stated that participants were explicitly instructed to use both visual and kinesthetic motor imagery. However, did the authors ask participants which modality they actually used to imagine the movement? Furthermore, since a total score (VMI + KMI) was calculated, do we know which modality was preferentially used by the participants?

Thank you for this question. Unfortunately, we did not ask participants which motor imagery modality they used during the protocol or which modality they preferred. We will carefully take this suggestion into account for a future study on the effects of unpleasant stimuli on motor imagery vividness.

Lines 106-107 → "Students and staff of the University of Montpellier were recruited to participate." Did the participants have any prior experience with motor imagery?

Indeed, this suggestion is particularly relevant. Participants were not interviewed about their prior experience with motor imagery training. However, during the completion of the KVIQ (Kinesthetic and Visual Imagery Questionnaire), and because the first author was the investigator, it was evident that all participants were unfamiliar with the concept of motor imagery. This was clear as all participants required time for an explanation of the concept. We can assure you that all participants were not familiar with motor imagery. Unfortunately, we did not include this criterion in the eligibility requirements.

Finally, please note that university staff were initially invited to participate in this study. However, only students were included, so the reference to staff was deleted from the manuscript.

Comment 3. Regarding the wrist movements, with which hand are they performed physically and/or imagined? It seems to me that this is not specified in your protocol. I assumed they were done with the left hand (figure 3)—could you please confirm?

We thank Reviewer 2 for their attention to the details of the protocol. All participants were right-handed, which is why we used the Edinburgh Handedness Inventory - Short Form (EHI-SF) to confirm this. In accordance with these suggestions, we have added the following sentence in the Methods section:

Line 149-151:” Since all participants were right-handed, the movement was executed and imagined using the dominant right hand, against gravity due to the action of the extensor muscles.”

This precision was added to the results section according to the EHI score.

Line 271-272:” According to the EHI laterality scores, all participants were right-handed.”

In addition, we have revised and updated the hand iconography in Figure 2 to ensure coherence.

Comment 4 The thermal stimulus. This brings me to another question regarding the thermal stimulus. The right hand was immersed in water up to the wrist joint — but for how long exactly? Was it immersed throughout the entire duration of the discrete modality (4 seconds × 20) and the continuous modality (3 × 25 seconds)? Was the hand removed from the water between blocks?"

Indeed, this important question about the protocol has been addressed in the protocol conditions in the Methods section as follows:

Line 213-217:” The initial duration of the hot water immersion was based on a study by Granot et al. (48), in which participants experienced a 60-second immersion. However, due to the length of our protocol and to minimize the risk of adverse events, we reduced the immersion time to 30 seconds before the tasks began.”

In both discrete and continuous sessions, the hand was immersed before each task, whereas the auditory stimulus was presented during the task. his detail has been added to Figure 2 for clarity.

Comment 5. Auditory stimulus. I also have a similar question regarding the auditory stimulus. It is stated that 'the sound was played for periods of 25 seconds through loudspeakers' — but when exactly did this occur? Was it during each block of the continuous modality, and was it repeated or paused between blocks?

I believe the authors should consider adding a legend to Figure 3 to clarify these details that appear to be missing, which are important for fully understanding the protocol.

Thank you for this suggestion. Indeed, the auditory stimulus was played during the tasks, whereas the heat immersion was conducted prior to the tasks. We have added this clarification in the figure 2 and in the Methods section:

Line 231-235:” For the thermal stimulus, the dominant hand was immersed in water up to the wrist joint before the task, and for the auditory stimulus, the sound was played through loudspeakers for 25-second periods during the task (Fig. 2). Participants were stopped at mid-movement to ensure that the movement was completed without acceleration or deceleration at the end of the continuous session.”

Comment 5. Results. There is an issue with the formatting regarding the results and the numbering of the tables. I noticed that the results for the discrete motor imagery modality appear twice: first between lines 254 and 255 (Table 2: normal median = 6.5; heat median = 6.0; auditory median = 4.5), and again between lines 280 and 281. I believe this is a duplication error. Please kindly correct the formatting, possibly by placing the tables at the end of the manuscript.

Indeed, we apologize for this oversight. We have made the necessary corrections and provided additional tables to enhance readability.

If I’m not mistaken, here is the proposed numbering for the tables:
- Table 1: Descriptive characteristics of the participants
- Table 2: Summary of the Total MIQ-RS score in each condition during the discrete motor imagery modality
- Table 3: Summary of the Total MIQ-RS score in each condition during the continuous motor imagery modality

Thank you for your response. We have implemented the suggested changes and consolidated Tables 2 and 3.

Furthermore, you state that you used the KVIQ scales as the primary outcome measure. However, in the manuscript, the tables are labeled with "Total MIQ-RS" scores. This inconsistency is confusing. Could you please clarify this point: did you use the KVIQ or the MIQ-RS?

We apologize for this oversight. This error occurred because we reused old R code from another study that used the MIQ-RS. We did indeed use the KVIQ questionnaire. The necessary changes have been made.

And, please correct the reference error on line 275: (Error! Reference source not found).

Indeed, we apologize for this error due to figure referencing in the Word document. The necessary changes have been made.

7. PLOS authors have the option to publish the peer review history of their article ([what does this mean?](https://journals.plos.org/plosone/s/editorial-and-peer-review-process#loc-peer-review-history)). If published, this will include your full peer review and any attached files.


If you choose “no”, your identity will remain anonymous but your review may still be made public.


**Do you want your identity to be public for this peer review?** For information about this choice, including consent withdrawal, please see our [Privacy Policy](https://www.plos.org/privacy-policy).

Reviewer #1: **Yes:** Sébastien MATEO

Reviewer #2: No
